# Unleashing the biological potential of marine algal extracts against* Staphylococcus aureus* isolated from ready-to-eat beef products

**DOI:** 10.1038/s41598-025-14674-w

**Published:** 2025-08-17

**Authors:** Mohamed Korashe Dandrawy, Tufahah M. O. Atiyahullah, Hani Saber, Ghada Hadad, Manar M. Abdelaleem, Hassan Mahmoud Diab, Hanan H. Abdelhafeez, Ahmed Shaban Ahmed, Mona A. El-Zamkan, Nady Khairy Elbarbary

**Affiliations:** 1 Department of Food Hygiene and Control (Meat Hygiene), Faculty of Veterinary Medicine, South Valley University, Qena, 83523 Egypt; 2 Department of Food Hygiene, Faculty of Veterinary Medicine, Omar ALmukhtar University, P.O. Box 919, Elbeida, Libya; 3 Department of Botany and Microbiology, Faculty of Science, South Valley University, Qena, 83523 Egypt; 4 Department of Animal Hygiene and Zoonoses, Faculty of Veterinary Medicine, University of Sadat City, Sadat City, 32897 Egypt; 5 Department of Fish Health and Diseases, Faculty of Fish and Fisheries Technology, Aswan University, Aswân, 81528 Egypt; 6 Department of Animal and Poultry Health and Environment, Faculty of Veterinary Medicine, South Valley University, Qena, 83523 Egypt; 7 Department of Cell and Tissues, Faculty of Veterinary Medicine, Assiut University, Assiut, 71526 Egypt; 8 Department of Food Hygiene and Control (Milk Hygiene), Faculty of Veterinary Medicine, South Valley University, Qena, 83523 Egypt; 9 Department of Food Hygiene and Control, Faculty of Veterinary Medicine, Aswan University, Aswân, 81528 Egypt

**Keywords:** *Staphylococcus aureus*, Meat products, Biofilm formation, Antimicrobial resistance, Antimicrobial bioactivity, Algal extracts, Antibiotics, Antimicrobial resistance, Microbiology, Antimicrobials, Applied microbiology, Biofilms, Microbial communities, Microbial genetics

## Abstract

*Staphylococcus aureus* is the causative agent of zoonotic diseases that are of increasing epidemiological importance and have significant implications for public health. One hundred fifty ready-to-eat meat product samples were collected from local shops in Qena City, Egypt, to investigate the bacteriological profile of *S. aureus* and evaluate the antimicrobial effects of marine algal extracts against identified *S. aureus* isolates in vitro. *S. aureus* was detected in 30.7% of the samples examined, with the highest incidence in luncheon samples. Detection of virulence genes revealed that 58.7%, 47.8%, 0%, 13%, and 0% of *S. aureus* isolates harbored *coa*, *nuc*, *sea*, *seb*, and *sec* genes. *S. aureus* isolates demonstrated a high level of antimicrobial resistance. Genetic analysis of antimicrobial resistance determinants revealed that 65.2% of the isolates carried the *mec*A gene, 72.2% harbored the *van*A gene, and 33.3% contained the *optr*A gene. These findings indicate a significant prevalence of genetic markers associated with resistance to methicillin, vancomycin, and oxazolidinones, respectively, among the studied *S. aureus* isolates. In addition, these isolates produced various biofilm phenotypes. The most-produced biofilm phenotype was strong (32.6%). Luncheon meat showed the highest MRSA confirmation rate (36.4%), followed by sausage (37.5%), while burger products had the lowest confirmation rate (25.0%). *Halimeda opuntia*, *Jania rubens*, and *Caulerpa racemosa* marine algal extracts’ antimicrobial activity was studied. The major constituent of *Caulerpa racemosa* extract was spathulenol, and the methanol extract of *Caulerpa racemosa* (1.5 mg/ml) exhibited the strongest antimicrobial activity against *S. aureus*. RT-PCR revealed significant downregulation of *coa* (98.7% reduction) and *nuc* (68.8% reduction) in *Caulerpa racemosa* extract-treated *S. aureus*. Algal extracts present a hopeful prospect for developing innovative antibacterial substances with great promise for application in food preservation and medicine.

**Supplementary Information:**

The online version contains supplementary material available at 10.1038/s41598-025-14674-w.

## Introduction

*Staphylococcus* genus comprises numerous species and subspecies, with *Staphylococcus aureus* (*S. aureus*) being a significant foodborne pathogen^1^. *S. aureus* is a Gram-positive bacterium that lacks motility and spore formation. It has a round shape and is facultative, anaerobic, and toxic. Commonly found in the environment and on the skin, nostrils, and respiratory systems of humans and animals, *S. aureus* causes food poisoning and a wide range of infections—from skin and soft tissue infections to severe illnesses^1^. It ranks among the most prevalent foodborne pathogens responsible for intoxication. Staphylococci can enter food processing environments through various routes, including raw materials, food handlers, and inadequate hygiene in processing equipment^2^.

*Staphylococcus aureus* rapidly multiplies at room temperature, leading to the production of toxins responsible for food poisoning. These heat-stable toxins, known as staphylococcal enterotoxins (S.E.), can cause illness when ingested through contaminated food. The most commonly involved food items are meat, meat products, milk and dairy products, bakery products, salads, etc.^2^. Regarding prevalence and frequency, staphylococcal intoxications rank third globally among all foodborne diseases^3^. Because of the growing number of *S. aureus* isolates expressing a broad spectrum of antibiotic resistance and virulence characteristics, the problem of food intoxication became a greater challenge, especially in ready-to-eat (RTE) food, which does not require to be cooked before consumption and serves as a vector for transmitting antibiotic-resistant microbes^4^.

The extensive use of antibiotics in bacteria contributes to the rise of multiple drug-resistant (MDR) strains, presenting significant obstacles to public health. *S. aureus* can adapt to different environmental conditions and quickly develop resistance to nearly all antibiotics. Methicillin-resistant *S. aureus* (MRSA) is a significant emerging nosocomial pathogen, and it was isolated from food and caused outbreaks of food poisoning^5^. Additionally, biofilms produced by *S. aureus* can provide protection against antibiotics, host immune system enzymes, and bad environmental conditions^6^.

MRSA is responsible for the largest outbreak of hospital-acquired infections (HAI) that the world has ever seen, with double the mortality of methicillin-susceptible *S. aureus* (MSSA) infections. The pathogen causes severe morbidity and mortality in hospitals worldwide, with incidence rates ranging from 0.71 to 10.0 per 1000 hospital admissions^7^.

The presence of methicillin-resistant *Staphylococcus aureus* (MRSA) in ready-to-eat meat products represents a significant One Health concern, as these food items bypass essential cooking steps that would typically eliminate pathogenic bacteria. This unique risk facilitates direct transmission of MRSA from the food chain into healthcare environments through multiple pathways^8,9^.

One primary route of transmission is through patient consumption. Hospitalized individuals who ingest contaminated ready-to-eat meats may inadvertently introduce MRSA into clinical settings, potentially leading to colonization or infection. Additionally, healthcare workers and hospital visitors who consume such products outside or within healthcare facilities may become asymptomatic carriers, increasing the risk of nosocomial spread. Furthermore, food-service areas within hospitals can become reservoirs for environmental contamination, serving as additional vectors for MRSA dissemination among vulnerable patient populations ^10,11^.

Livestock-associated MRSA (LA-MRSA) also plays a critical role in the broader epidemiology of MRSA. The food production system contributes to its dissemination through several factors. Notably, the use of antimicrobials in animal husbandry applies selective pressure, promoting the emergence and persistence of resistant strains. Contamination during slaughter and subsequent food processing stages further amplifies the risk, especially when sanitary practices are inadequate. Cross-contamination between animal and human MRSA strains-facilitated by poor hygiene and handling-exacerbates the challenge, highlighting the need for integrated control measures across the farm-to-fork continuum^9,11^.

Natural preservatives are in high demand for both food safety and quality. Algae include a wide range of natural bioactive chemicals with antibacterial properties. As a result, they may offer promising alternatives for medical therapy as well as novel natural antimicrobial compounds to substitute synthetic antibacterial compounds applied in agriculture and the food sector^12^. Several investigations have demonstrated algae’s antibacterial activity regarding microbes^13^. Therefore, algae extracts might be used as natural preservatives in foods to improve their quality, safety, and longevity, which might be a promising substitute for physical and chemical techniques^14^.

The research aimed to investigate the occurrence of *Staphylococcus* species, particularly *S. aureus*, in RTE meat product samples from Qena City, Egypt. Additionally, it examined the presence of specific virulence genes coagulase (*coa*), thermonuclease (*nuc*), and enterotoxins (S.E.s; *sea*, *seb*, and *sec*) in *S. aureus* isolates. The study assessed the antibiotic resistance profiles of these strains, including genes associated with antimicrobial resistance (*mec*A, *van*A, and *optr*A). Furthermore, it explored the biofilm-forming ability of isolated strains and examined biofilm-related genes (*ica*A and *ica*D). Moreover, the research evaluated the antimicrobial effects of marine algal extracts against identified *S. aureus* isolates in vitro. Furthermore, investigate the inhibitory effects of *Caulerpa racemosa* extract on *S. aureus* virulence genes (*coa* and *nuc*) using RT-PCR. Novel techniques are necessary to meet the targeted demographic’s health concerns adequately. The current study’s results and suggestions will prompt critical health decision-makers to adopt suitable countermeasures.

## Materials and methods

### Collection of samples

A total of 150 samples, including RTE beef products of shawarma, kofta, burgers, luncheon, and sausages (30 samples each), were randomly procured from fast-food restaurants in Qena City, Egypt from October 2023 until March 2024. Upon purchase, they were transferred to the central laboratory of Faculty of Veterinary Medicine, South Valley University in sterile plastic bottles with screw-top and refrigerated at (4 °C) for further analysis.

### Isolation and identification of *S. aureus*

Approximately 25 g of each sample was aseptically transferred to a sterile stomacher bag and homogenized with 225 ml of 0.1% sterile buffered peptone water. This mixture was thoroughly blended, resulting in a tenfold serial dilution following APHA^15^. 0.1 ml of each of the prepared serial dilutions was inoculated onto a duplicate Baird Parker agar plate, supplemented with egg-yolk tellurite emulsion, and incubated for 48 h at 37 °C.

Presumptive *S. aureus* isolates were confirmed according to morphology and biochemical characteristics including hemolysis testing following overnight incubation at 37 °C on sheep blood agar. The agar was prepared with 15 ml of 5% sheep blood in Trypticase soy agar (Becton Dickinson) overlaid on 10 ml of blood agar base (Oxoid Ltd., Basingstoke, Hampshire, United Kingdom). Hemolysis types were classified as α-hemolysis, β-hemolysis, double hemolysis (α + β), or negative (no hemolysis)^16,17^.

DNase activity was evaluated on DNase test agar according to the manufacturer’s protocol (Difco Laboratories, Detroit, Mich.). Positive DNase activity was identified by a clear zone surrounding the growth, whereas weak activities, producing smaller clearing zones than the positive control, were considered negative. The coagulase test involved using rabbit plasma, with results recorded after 24 h of incubation at 37 °C; even weak coagulase activities were noted as positive. The production of a yellow-to-orange pigment indicated Staphyloxanthin production, confirming the isolates as *S. aureus*^18^.

### Antibiogram pattern of *S. aureus*

Fifteen antimicrobials belonging to 11 antibiotic groups were tested against all *S. aureus* strains following the methodology outlined by Bauer et al.^19^. Discs of Ampicillin (AM;10 μg); Penicillin G (P; 10 units); Oxacillin (OX; 1 μg); Amikacin (AK; 30 μg); Chloramphenicol (C; 30 μg); Ciprofloxacin (CIP; 5 μg); Cefotaxime (FOX; 30 μg); Kanamycin (K; 30 μg); Erythromycin (E; 15 μg); Nalidixic acid (NA; 30 μg); Norfloxacin (NOR; 10 μg); Trimethoprim/ Sulphamethoxazol (SXT; 1.25/23.75 μg); Tetracycline (TE; 30 μg); Vancomycin (VA; 5 μg); and Linezolid (LZD; 30 μg) were used and obtained from Oxoid, UK. CLSI determined the results^20^.

### Detection of biofilm formation by the microplate (M.P.) method

The evaluation of biofilm formation through the M.P. method was conducted based on the methodology outlined by Stepanović et al.^21^ using a sterile 96-well flat-bottomed polystyrene microtiter plate (Nunc) for the assessment. Each plate well was filled with 200 µl of sterile BHI broth (Merck Millipore). In triplicate, 20 µl of overnight *S. aureus* cultures (1 × 10^9^ cells/ml) were added to each well. Wells designated negative controls were filled with sterile broth that had not been inoculated. The plates were incubated at a temperature of 30 °C for 24 h. Then, the bacterial suspension was carefully aspirated, and each well underwent a thorough rinsing three times with 250 µl of PBS (Sigma). Subsequently, 200 µl of 99% ethanol was used to fix the formed biofilm for 15 min, after which it was removed. The plates were then air-dried at ambient temperature and subjected to staining using a 200 µl crystal violet solution for 5 min. Following staining, the plates were thoroughly rinsed under running water until any unbound crystal violet was completely removed, and the plates were air-dried again at room temperature. To remove the dye from the adherent cells, 160 µl of 33% (v/v) glacial acetic acid was introduced into each well. To quantify the formation of biofilm, the dissolution of adherent cells was done using glacial acetic acid (33%). The Optical densities (O.D.) were measured using a plate reader at a wavelength of 570 nm. The resulting O.D. of each strain was then compared to the cut-off of (ODc), which is calculated as three standard deviations above the mean O.D. of the negative control. To categorize the extent of biofilm formation, the following criteria were employed: no biofilm production (O.D. ≤ ODc), weak biofilm production (ODc < O.D. ≤ 2ODc), moderate biofilm production (2ODc < O.D. ≤ 4ODc), and strong biofilm production (4ODc < O.D.).

### Detection of virulence, antibiotic resistance, and biofilm-encoding genes in *S. aureus* strains

Molecular detection of five virulence genes, namely *coa* (coagulase gene), *nuc* (thermonuclease), *sea*, *seb*, and *sec*, was performed using the PCR technique according to Mehrotra et al.^22^. Additionally, the presence of *mec*A, *van*A and *optr*A genes that encode methicillin, vancomycin and linezolid resistance, respectively were investigated. Moreover, the presence of biofilm-encoding genes *ica*A and *ica*D was screened^23–28^. The primer sequences, amplicon size, and PCR programs are listed in (Supplementary Table S1). DNA extraction was done following the instructions provided by the QIAamp DNA Mini kit instructions (Qiagen, Germany, GmbH). The amplification was done using the Emerald Amp GT PCR master mix (Takara) Code No. RR310A (2X), and the resulting products were analyzed through agarose gel electrophoresis.

### Antimicrobial activity of algal extracts against *S. aureus*

#### Microorganisms

Virulent strains of recovered *S. aureus* ATCC strain (25,923) (confirmed and obtained from Bacteriology Department and Food Hygiene Department, Animal Health Research Institute, Cairo, Egypt) were enriched in tryptic soy broth. Subsequently, they were cultivated on a Baird Parker agar plate, supplemented with egg-yolk tellurite emulsion at 28–30 °C for 24 h before assay^29^.

#### Algal collection and extraction preparation

In April, three marine algal species were collected from Hurghada, Egypt’s Red Sea coast: *Halimeda opuntia* (Chlorophyta), *Jania rubens* (Rhodophyta), and *Caulerpa racemosa* (Chlorophyta). Taxonomic identification followed standard keys by Aleem^30^, Dhargalkar and Kavlekar^31^. The samples were collected in sterilized polyethylene bags and kept in an ice box during transport to the laboratory. After thorough rinsing with sterile distilled water and air-drying, the sample was ground into fine powder consistency using an IKA A 10 tissue grinder (Germany). Different organic solvents (methanol, ethanol, acetone, ethyl acetate, and petroleum ether) were used for compound extraction. In the lab, 10 g of dried powdered samples were immersed in 100 ml of the solvent for five days at room temperature under stirring conditions (150 rpm). The extracts were filtered through Whatman No. 1 filter paper and dried using Cole-Parmer Instrument desiccators (Chicago). A final concentration of 50 mg/ml of the weighted crude extracts was suspended in the dimethyl sulfoxide (DMSO) in an airtight bottle and then stored in a refrigerator until experiment^13^.

#### Analysis of the algal extracts

Algal extracts were analyzed using the gas chromatography-mass spectrometry (GC–MS) technique. Thermo Scientific Technologies Trace 1310 instrument, equipped with capillary column TG-5 (30 m × 250 μm × 0.25 μm) system was used. A mass detector was operated in split mode, and helium gas was the carrier at a flow rate of 1.5 ml/min. The injector was operated at a temperature of 230 °C, and the oven temperature started at 60 °C for two min, ramped up at a rate of 10 °C per min to 300 °C for eight min. Mass spectra were examined at 70 eV, and the total running time for the G.C. was 35 min^12^.

#### Antimicrobial assay

The liquid-broth method^32^ was employed to evaluate the efficacy of various concentrations of algal extracts in controlling *S. aureus*. Bacterial cultures were cultivated in Brain Heart Infusion (BHI) broth until they reached the mid-logarithmic phase. The bacterial cells were washed and resuspended to achieve a 6–7 log10 CFU/ml concentration in 1% Trypticase Soy Broth (TSB) with a pH of 7.3^33^. 100 µl bacterial suspension was pipetted onto the wells of a 96-well microplate, which already contained 100 µl of algal extracts at varying concentrations (ranging from 0 to 1500 µg/ml). The microplate was incubated at 30 °C for 24 h, during which time the absorbance was measured from three parallel wells per sample at a wavelength of 620 nm (Supplementary Figure S1). The readings were background-corrected (subtracting the value obtained in the absence of bacteria). Finally, the obtained results represent data from three independent experiments.

#### Real-time PCR analysis (Yuan et al.)^34^

To evaluate virulence gene expression, *S. aureus* cultures (n = 10) were grown in TSB medium supplemented with *Caulerpa racemosa* extract (1.5 mg/ml) for 24 h at 37 °C. Cells were washed in PBS, standardized to ~ 2 × 10^9^ CFU/ml, and treated with mutanolysin (100 µg) and lysozyme (100 µg) for 15 min at 37 °C to prepare for RNA extraction. For RNA stabilization, 0.5 ml of bacterial broth was mixed with 1 ml RNA protect reagent (Qiagen), vortexed, incubated for 5 min at room temperature, and centrifuged (8000 rpm, 10 min). The pellet was resuspended in 200 µl TE buffer containing lysozyme (1 mg/ml), lysed with 700 µl RLT buffer (supplemented with β-mercaptoethanol), and homogenized with 500 µl ethanol. Total RNA was then purified using the QIAamp RNeasy Mini Kit, including on-column DNase digestion to eliminate genomic DNA contamination. The resulting RNA was reverse-transcribed into cDNA and analyzed via RT-PCR to compare gene expression between treated and untreated groups.

Gene-specific primers (Metabion) were used in 25 µl reactions containing: 12.5 µl 2 × QuantiTect SYBR Green Master Mix, 0.25 µl RevertAid Reverse Transcriptase (200 U/µl), 0.5 µl each primer (20 pmol), 6.25 µl water, and 5 µl RNA template. Reactions were run on a OneStep™ Real-Time PCR System with: 94 °C for 15 min; 40 cycles of 95 °C (15 s), 55 °C (30 s), 72 °C (30 s); and final extension at 72 °C for 10 min. Specificity was confirmed by melting curve analysis (55–95 °C). Data were analyzed using StepOne™ Software (v2.3), with gene expression calculated via the ΔΔCt method (ΔCt treated–ΔCt control).

### Statistical analysis

Statistical analysis using the Fisher’s exact test was performed by GraphPad Prism 8, and *p* < 0.05 was regarded as statistically significant.

## Result

### Bacteriological analysis of the samples

One hundred fifty samples of ready-to-eat (RTE) meat products, including shawarma, kofta, burger, luncheon, and sausage (30 samples of each product), were screened for the presence of *S. aureus.* Overall, 46 samples (30.7%) tested were positive for *S. aureus*. The highest occurrence % of *S. aureus* was found in luncheon (36.7%) with an average bacterial count of 6.1 × 10 ± 0.4 × 10 CFU/g. Following that, kofta (33.3%) had an average count of 2.1 × 10 ± 0.2 × 10 CFU/g, shawarma (30%) had an average count of 2.7 × 10 ± 0.3 × 10 CFU/g, and both burger and sausage (26.7%) had similar counts of 3.6 × 10 ± 0.7 × 10 CFU/g and 3.2 × 10 ± 0.5 × 10 CFU/g, respectively **(**Table 1**)**. The β- and α- hemolytic activities developed by *S. aureus* strains on blood agar plates after 48 h of culturing besides golden-carotenoid pigmentation colonies indicate Staphyloxanthin production and are recorded as positive for *S. aureus.*

**Table 1:** Statistical analytical results of *S. aureus* examined ready-to-eat meat products (N = 30).

Samples(N = 30)	Positive samples		*Staphylococcus aureus* count (cfu/g)		
	No	%	Minimum	Maximum	Mean ± S.E
Shawerma	9	30	1.70 × 10	6.7 × 10	2.7 × 10 ± 0.3 × 10
Kofta	10	33.3	1.1 × 10	4.3 × 10	2.1 × 10 ± 0.2 × 10
Burger	8	26.7	1.5 × 10	8.2 × 10	3.6 × 10 ± 0.7 × 10
Luncheon	11	36.7	4.6 × 10	8.9 × 10	6.1 × 10 ± 0.4 × 10
Sausage	8	26.7	1.9 × 10	7.8 × 10	3.2 × 10 ± 0.5 × 10
Total (150)	46				

### Incidence of virulence genes in isolated *S. aureus*

**Fig. 1:**
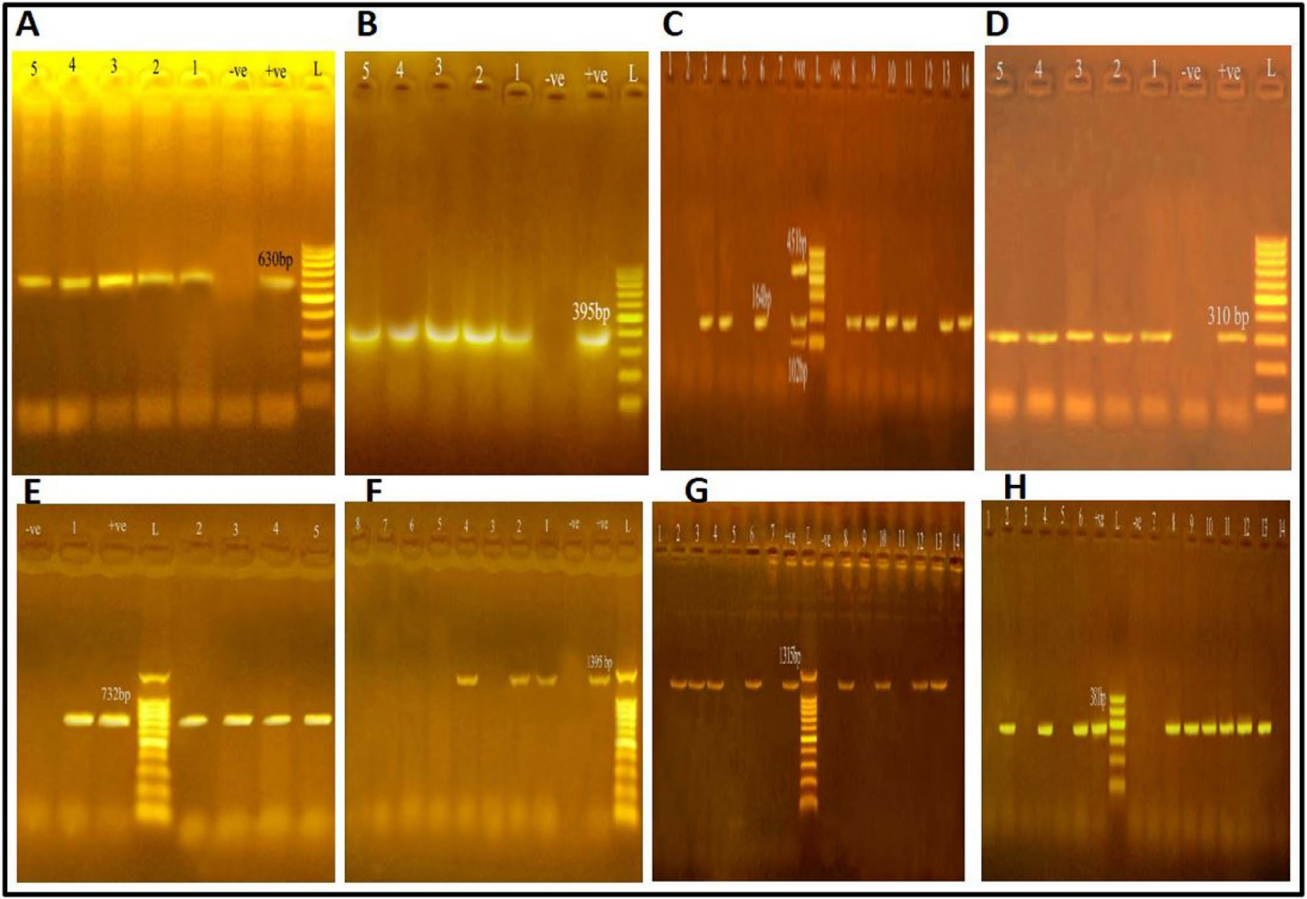
PCR products of amplified virulent genes and antibiotic resistance genes identified in *Staphylococcus aureus* visualized on agarose gel electrophoresis. The expected molecular size of amplified DNA: 630 bp for *coagulase* (*coa*) gene (**A**), 395 bp for *nuc* gene (**B**), 102 bp for *Sea* gene, 164 bp for *Seb* gene, 451 bp for *Sec* gene (**C**), 310 bp for *mec*A gene (**D**), 732 bp for *van*A gene (**E**), 1395 bp for *optr*A gene (**F**), 1315 bp for *ica*A gene (**G**), and 381 bp for *ica*D gene (**H**). Lane L: DNA ladder; DNA Ladder; 1–14: Sample; -ve: Negative control; + ve: Positive control.

The genes investigated encoding coagulase (*coa*), thermonuclease (*nuc*), and enterotoxins (*S.E.s*) (including *sea*, *seb*, and *sec*). The results showed that *coa* and *nuc* were present in 58.7% and 47.8% of the samples, respectively, while *S.E.s* (*sea*, *seb*, and *sec*) were 0%, 13%, and 0%, respectively (Table 2 and Fig. 1).

**Table 2:** Virulence genes profile of *S. aureus* strains screened by PCR.

Samples (N = 30)	Total No. of* Staphylococcus aureus*	Virulence genes									
		*coa*		*nuc*		*S.E.s*					
						*sea*		*seb*		*sec*	
		No	%	No	%	No	%	No	%	No	%
Shawerma	9	5	55.6	4	44.4	0	0	1	11.1	0	0
Kofta	10	5	50.0	4	40.0	0	0	1	10.0	0	0
Burger	8	5	62.5	5	62.5	0	0	1	12.5	0	0
Luncheon	11	7	63.6	6	54.5	0	0	2	18.2	0	0
Sausage	8	5	62.5	3	37.5	0	0	1	12.5	0	0
Total (150)	46	27	58.7	22	47.8	0	0	6	13.0	0	0

### Antibiogram pattern of *S. aureus* isolates

**Fig. 2:**
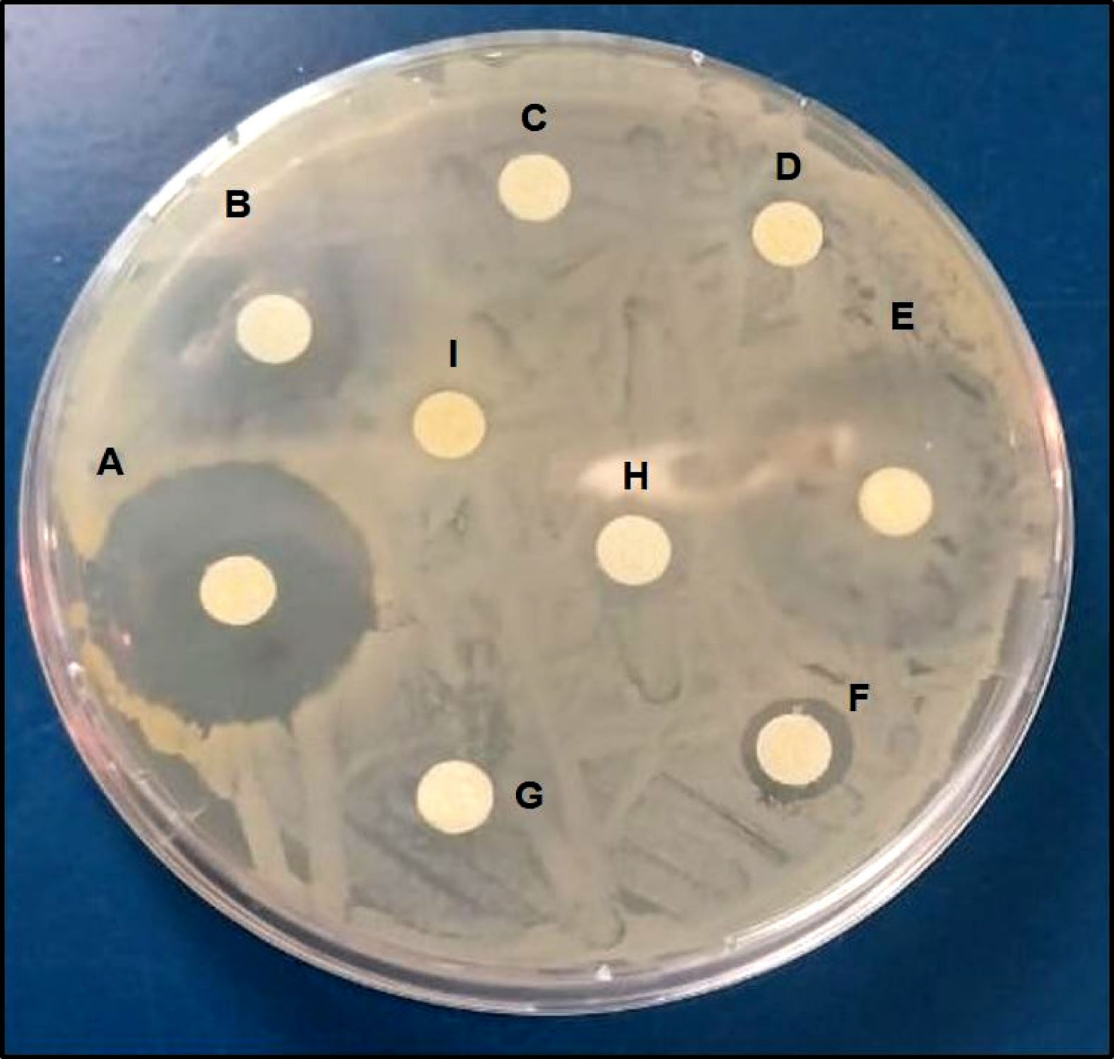
Disc diffusion test; from A-H samples of disc diffusion test for (A) Norfloxacin (NOR; 10 μg); (B) Linezolid (LZD; 30 μg); (C) Kanamycin (K; 30 μg); (D) Chloramphenicol (C; 30 μg); (E) Ciprofloxacin (CIP; 5 μg); (F) Cefoxitin (FOX; 30 μg); (G) Penicillin G (P; 10 units); (H) Oxacillin (OX; 1 μg); (I) Nalidixic acid (NA; 30 μg).

**Fig. 3:**
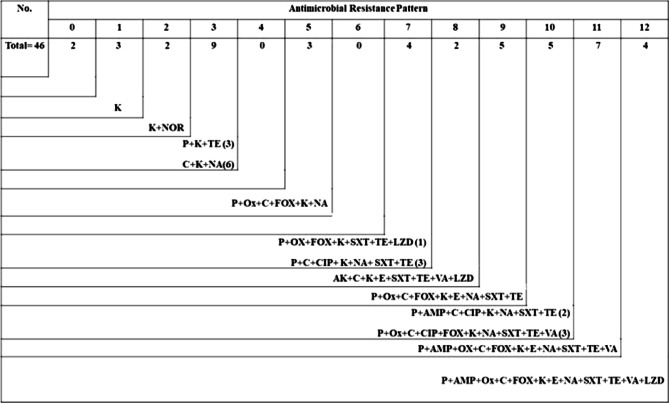
Antimicrobial resistance pattern of *Staphylococcus aureus* isolates obtained from the examined samples. Ampicillin (AM;10 μg); Amikacin (AK; 30 μg); Penicillin G (P; 10 units); Oxacillin (OX; 1 μg); Kanamycin (K; 30 μg); Chloramphenicol (C; 30 μg); Ciprofloxacin (CIP; 5 μg); Norfloxacin (NOR; 10 μg); Cefoxitin (FOX; 30 μg); Erythromycin (E; 15 μg); Nalidixic acid (NA; 30 μg); Sulfamethoxazole/Trimethoprim (SXT; 1.25/23.75 μg); Tetracycline (TE; 30 μg); Vancomycin (VA; 5 μg); Linezolid (LZD; 30 μg).

**Fig. 4:**
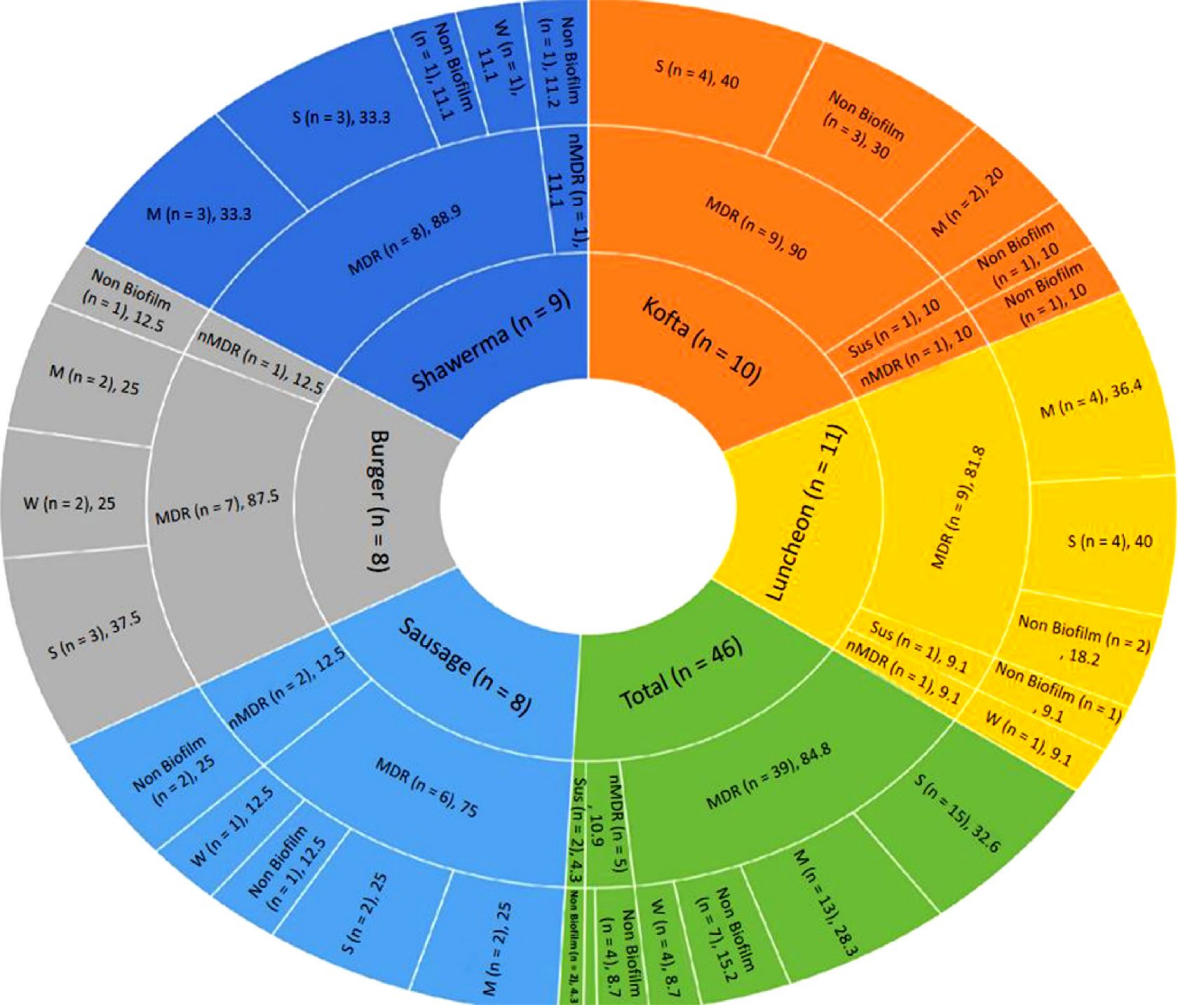
Incidence of MDR *Staphylococcus aureus* in different examined samples and biofilm types in the obtained isolates.

The antimicrobial susceptibilities of 46 *S. aureus* isolates were evaluated for 15 commonly used antimicrobial agents in veterinary clinics and farms. Irrespective of their source, the examined isolates demonstrated resistance to Penicillin G (67.4%), Ampicillin (28.3%), Oxacillin (50%), Amikacin (4.3%), Kanamycin (95.7%), Chloramphenicol (76.1%), Ciprofloxacin (19.6%), and Norfloxacin (4.3%). They were, however, susceptible to other antimicrobials such as Cefoxitin (50%), Erythromycin (37%), Nalidixic acid (71%), Sulfamethoxazole/Trimethoprim (58.7%), Tetracycline (65.2%) Vancomycin (39.1%), and linezolid (19.6%) (Table 3, Fig. 2). As shown in (Fig. 3), 11 antimicrobial resistance patterns were observed in isolated *S. aureus* strains. Of the 46 isolates, 39 were MDR (84.8%) (Fig. 4). The antimicrobial *mec*A, *van*A*,* and *optr*A resistance genes were detected in 65.2, 72.2, and 33.3% of phenotypic methicillin, vancomycin, and linezolid-resistant *S. aureus* isolates, respectively (Table 4).

**Table 3:** Antibiogram resistance pattern of *S. aureus* isolates.

Antibiotic group	Antibiotic	Samples (No. of isolates)					
		Shawarma (9) No. (%)	Kofta (10) No. (%)	Burger (8) No. (%)	Luncheon (11) No. (%)	Sausage (8) No. (%)	Total (46) No. (%)
Penicillins	Penicillin G	6 (66.7)^b^	6 (60)^b^	6 (75)^ab^	8 (72.7)^ab^	5 (62.5)^ab^	31 (67.4)^b^
	Ampicillin	3 (33.3)^bc^	3 (30)^bc^	3 (37.5)^bc^	2 (18.2)^c^	2 (25)^bc^	13 (28.3)^bc^
	Oxacillin	5 (55.6)^b^	5 (50)^b^	4 (50)^bc^	5 (45.5)^b^	4(50)^b^	23 (50)^c^
Aminoglycosides	Amikacin	0 (0)^d^	1 (10)^c^	0 (0)^d^	1 (9.1)^c^	0 (0)^d^	2 (4.3)^d^
	Kanamycin	9 (100)^a^	9 (90)^a^	8 (100)^a^	10 (90.9)^a^	8(100)^a^	44 (95.7)^a^
Phenicols	Chloramphenicol	7 (77.8)^ab^	9 (90)^a^	5 (62.5)^b^	9 (81.8)^a^	5(62.5)^ab^	35 (76.1)^ab^
Fluoroquinolones	Ciprofloxacin	1 (11.1)^c^	2 (20)^c^	1 (12.5)^c^	3 (27.3)^bc^	2(25)^bc^	9 (19.6)^c^
	Norfloxacin	0 (0)^d^	0 (0)^d^	0 (0)^d^	1 (9.1)^c^	1 (12.5)^c^	2 (4.3)^d^
Cephalosporins	Cefoxitin	5 (55.6)^b^	5 (50)^b^	4 (50)^b^	5 (45.5)^b^	4 (50)^b^	23 (50)^c^
Macrolides	Erythromycin	3 (33.3)^bc^	4 (40)^bc^	3 (37.5)^bc^	5 (45.5)^b^	2 (25)^bc^	17 (37)^bc^
Quinolone	Nalidixic acid	7 (77.8)^ab^	8 (80)^ab^	5 (62.5)^b^	8 (72.7)^ab^	5 (62.5)^ab^	33 (71.7)^ab^
Folate Pathway	Sulfamethoxazol/	5 (55.6)^b^	6 (60)^b^	4 (50)^b^	8 (72.7)^ab^	4 (50)^b^	27 (58.7)^c^
Antagonists	Trimethoprim						
Tetracyclines	Tetracycline	5 (55.6)^b^	6 (60)^b^	6 (75)^ab^	8 (72.7)^ab^	5 (62.5)^ab^	30 (65.2)^b^
Glycopeptides	Vancomycin	3 (33.3)^bc^	5 (50)^b^	4 (50)^b^	3 (27.3)^bc^	3 (37.5)^b^	18 (39.1)^bc^
Oxazolidinones	Linezolid	3 (33.3)^bc^	4 (40)^bc^	0 (0)^d^	1 (9.1)^c^	1 (12.5)^c^	9 (19.6)^c^

Values within a column with the same letters are not significantly different at *p* < 0.05.

**Table 4:** Antibiotic resistance encoding genes in the obtained *S. aureus* strains.

Samples	Antibiotic resistance genes (No. of isolates showed phenotypic resistance)					
	*mec*A (23)		*van*A (18)		*optr*A (9)	
	No	%	No	%	No	%
Shawerma	3	13	2	11.1	1	11.1
Kofta	3	13	3	16.7	1	11.1
Burger	2	8.7	3	16.7	0	0
Luncheon	4	17.4	3	16.7	1	11.1
Sausage	3	13	2	11.1	0	0
Total	15	65.2	13	72.2	3	33.3

### Phenotypic and genotypic characterization of biofilm formed by *S. aureus* isolates

**Fig. 5:**
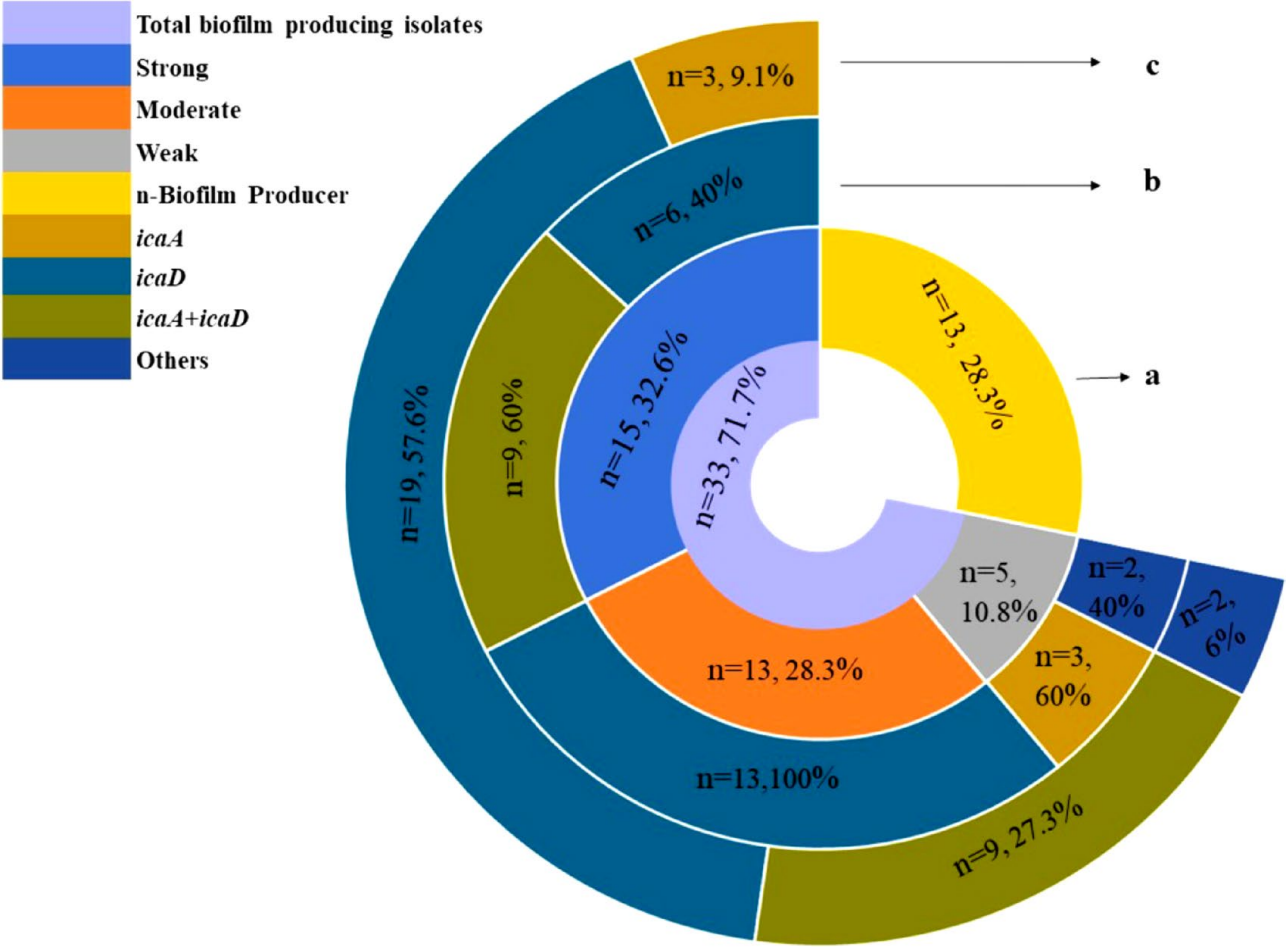
Biofilm-encoding genes in relation to phenotypic biofilm formation of *Staphylococcus aureus* isolates obtained from the examined samples. a: the incidence of different biofilm phenotypes detected in the examined *S. aureus* isolates. b: the frequency distribution of biofilm genes in relation to biofilm phenotype. c: the total incidence of biofilm-encoding genes detected in the biofilm-producing isolates.

**Fig. 6:**
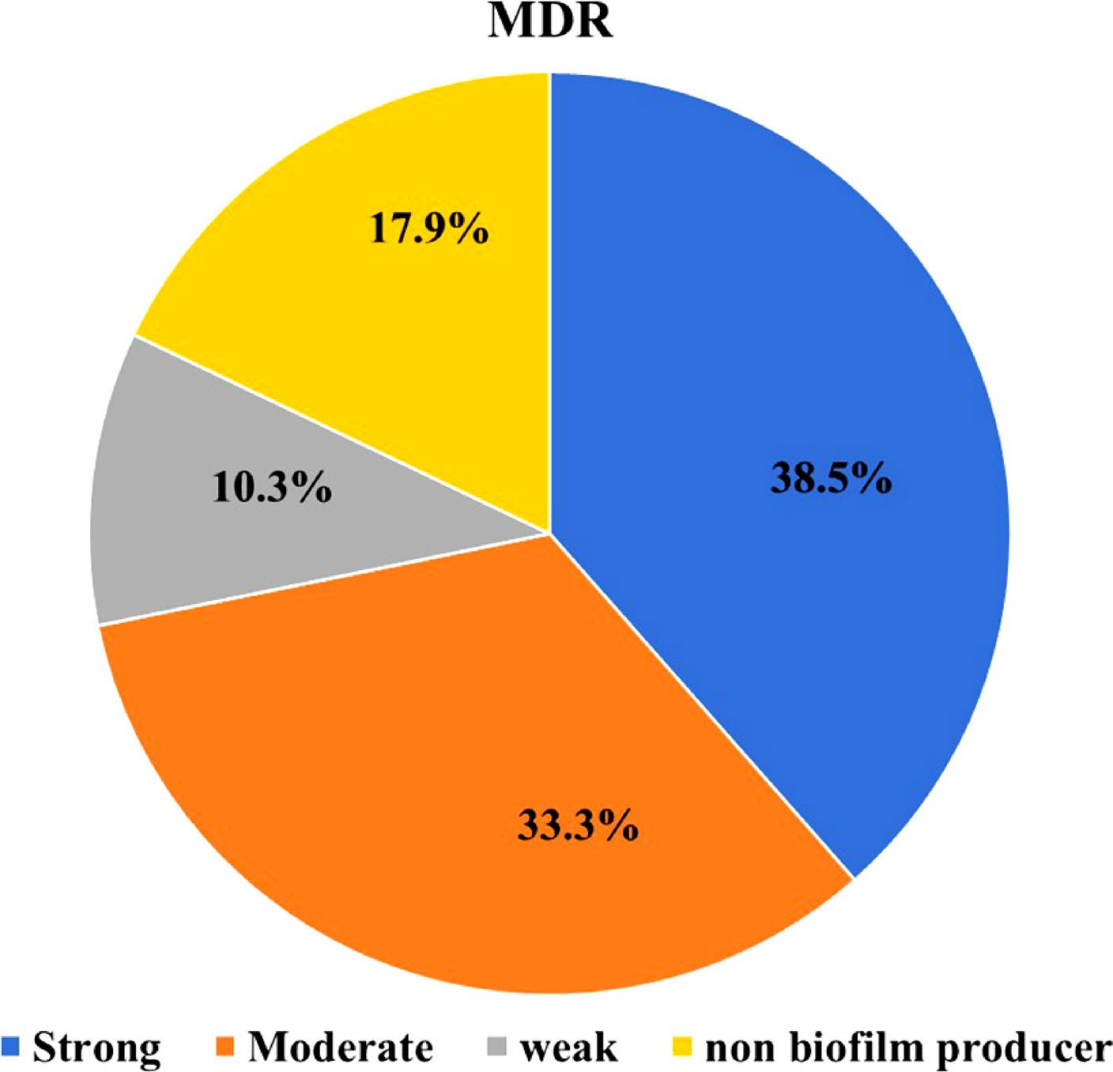
Distribution of different biofilm types in MDR *S. aureus* isolates.

Out of the 46 strains of *S. aureus*, 33 (71.7%) showed positive results for biofilm formation, as demonstrated in. It is noteworthy that among these strains, 15 were categorized as strong biofilm formers (32.6%), while 13 (28.3%) and five (10.9%) strains were moderate and weak biofilm formers, respectively. Furthermore, 13 (28.3%) strains did not produce any biofilm at all (Fig. 5a). Out of 39 MDR *S. aureus* isolates, 32 (82.1%) strains were biofilm producers (Fig. 4), 38.5%, 33.3%, and 10.3% of MDR *S. aureus* isolates produced strong, moderate, and weak biofilm (Fig. 6). There is a high significance between biofilm formation and drug resistance (*p* < 0.05).

The study focused on screening the *S. aureus* isolates to determine the overall distribution of biofilm-related *ica*A and *ica*D genes. Our findings confirmed that 31 (93.9%) biofilm-producing *S. aureus* isolates carry *ica*A or/and *ica*D genes. Additionally, isolates with just the *ica*D gene had the highest frequency (57.6%), while isolates with *ica*A + *ica*D had a frequency of 27.3%. On the other hand, isolates with just *ica*A had the lowest frequency (9.1%) (Fig. 5c). Furthermore, 40 and 60% of the strong biofilm-producing *S. aureus* strains carried *ica*D and *ica*A + D, respectively (Fig. 5b). While moderate and weak *S. aureus* biofilm producers carried *ica*A or *ica*D*,* respectively (Fig. 5b). There is a strong relation between the presence of both patterns of *ica*D and *ica*A + D genes and biofilm formation (*p* < 0.05).

### Identification and characterization of methicillin-resistant *S. aureus* (MRSA)

The confirmation of MRSA requires multiple diagnostic steps, each with specific significance in the identification process. 47.8% (22/46) of isolates carried the *nuc* gene, which encodes thermonuclease and serves as a species-specific marker for *S. aureus*. The *nuc* gene is widely used for molecular identification and confirms the virulence potential of the isolates. Furthermore, 32.6% (15/46) of isolates tested positive for the *mecA* gene, which is the gold standard for MRSA confirmation. The *mec*A gene encodes penicillin-binding protein 2a (PBP2a), which has low affinity for β-lactam antibiotics and is responsible for methicillin resistance^35^. Moreover, 50.0% (23/46) of *S. aureus* isolates demonstrated cefoxitin resistance, representing the initial phenotypic screening step. Cefoxitin is the preferred screening antibiotic because it is a more potent inducer of the *mec*A regulatory system than oxacillin and correlates better with the presence of the *mec*A gene^36^. Additional antibiotic resistance patterns vancomycin resistance: 39.1% of isolates (18/46) Linezolid resistance: 19.6% of isolates (9/46), these resistance patterns provide supplementary evidence of multidrug resistance, with vancomycin and linezolid representing last-line therapeutic options for MRSA infections. Luncheon meat showed the highest MRSA confirmation rate (36.4%), followed by sausage (37.5%), while burger products had the lowest confirmation rate (25.0%) despite having the highest *nuc* gene prevalence (62.5%) (Supplementary Table S2).

### Antimicrobial activity of the algal extracts

**Fig. 7:**
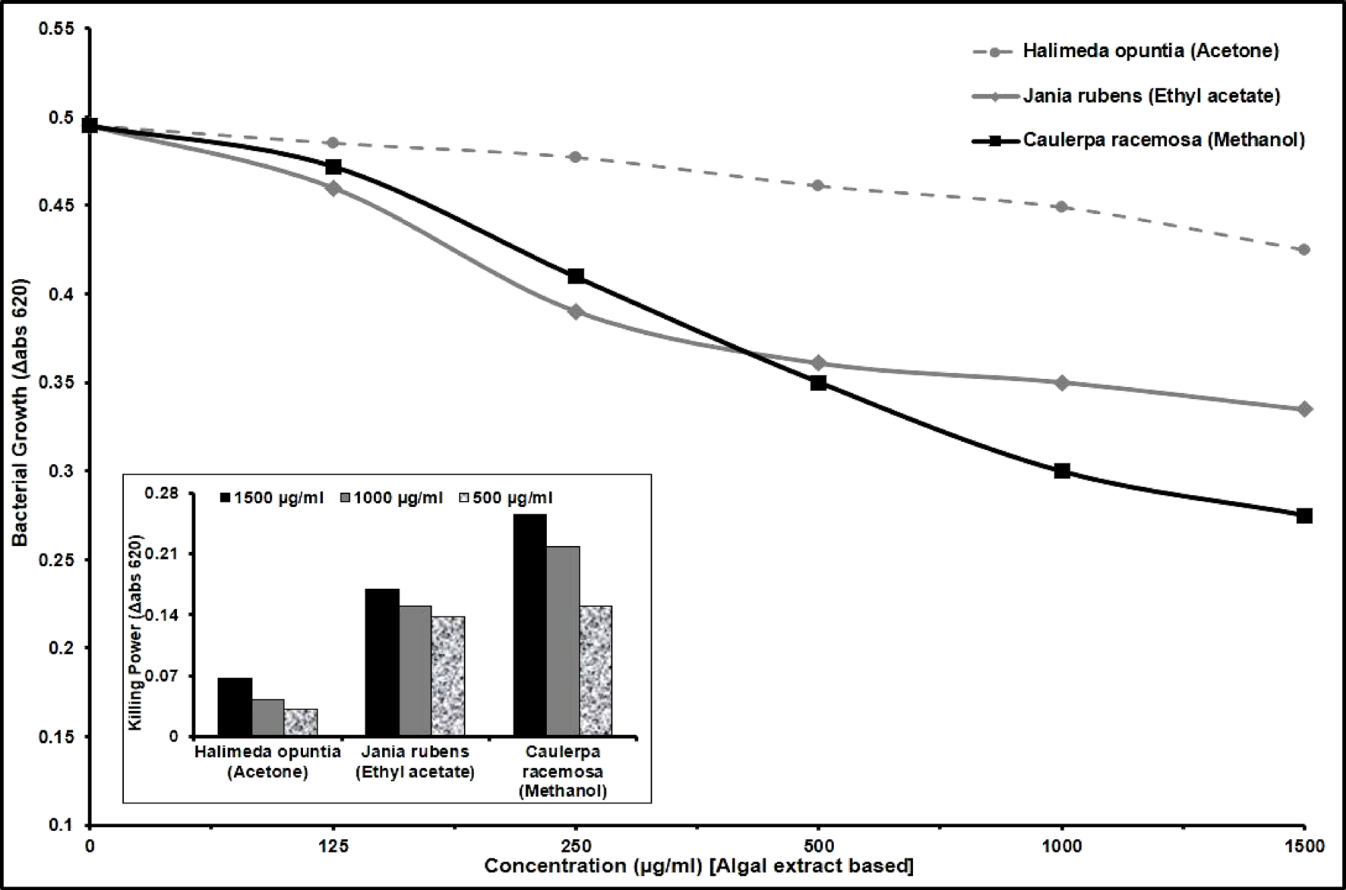
Antibacterial activity of algal extracts against *Staphylococcus aureus* at different concentrations. The data is presented as bacterial growth monitored at 620 nm. (Inset) Killing power of algal extracts (1500, 1000, and 500 µg/ml) against *Staphylococcus aureus* as bacterial growth monitored at 620 nm. The assays were performed in triplicate.

Three marine algal species, namely *Halimeda opuntia, Jania rubens*, and *Caulerpa racemosa* were assessed for their antimicrobial activity against recovered *S. aureus* isolates using a bactericidal assay method that measured bacterial growth at 620 nm, as shown in Fig. 7, Supplementary Table S3 and Supplementary Table S4.

*H. opuntia* (acetone extract), *J. rubens* (ethyl acetate extract), and *C. racemosa* (methanol extract) demonstrated potential effectiveness in inhibiting *S. aureus* growth in a dose-dependent manner with varying potency. Their activity was quantified by monitoring bacterial growth at 620 nm over a 24 h incubation period with *S. aureus*.

The methanol extract of *C. racemose* exhibited significant efficacy against *S. aureus*, resulting in a severe reduction in bacterial growth (Δabs 620) at a concentration of 1.5 mg/ml. In contrast, the ethyl acetate extract of *J. rubens* and the acetone extract of *H. opuntia* showed a lesser reduction in bacterial growth (Δabs 620) at the same concentration of 1.5 mg/ml, as depicted in Fig. 7, Supplementary Table S3 and Supplementary Table S4.

#### GC–MS analysis revealed the presence of specific compounds in each algal extract

**Fig. 8:**
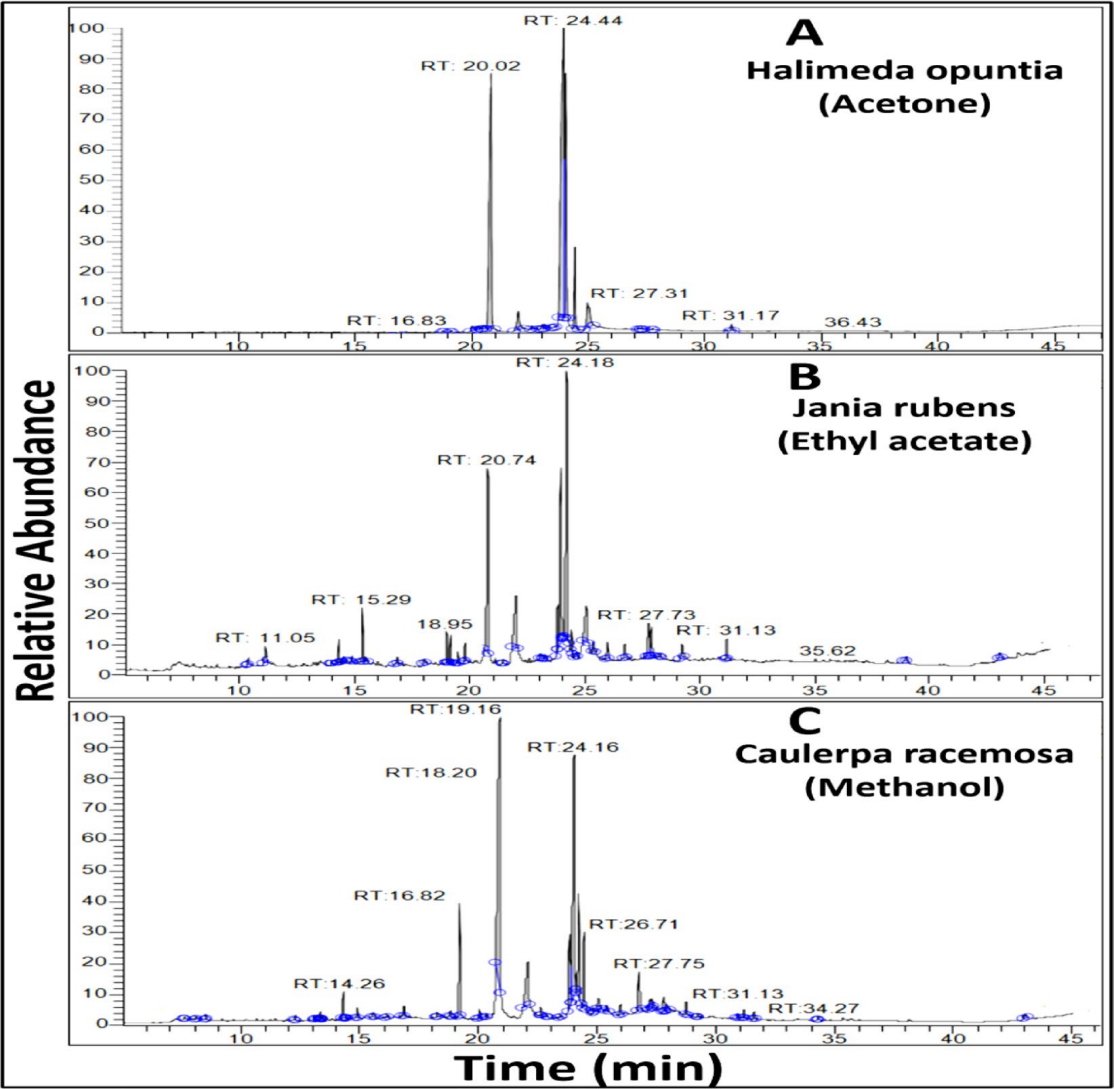
GC–MS chromatogram analysis of the most effective extracted constituents of algae: (**A**) *Halimeda opuntia* (extracted by acetone), (**B**) *Jania Rubens* (extracted by ethyl acetate), and (**C**) *Caulerpa racemosa* (extracted by methanol).

In *H. opuntia* (acetone extract), the most prevalent compounds included 9,12-Octadecadienoic acid (Z, Z)-, methyl ester (42.35%), Hexadecanoic acid, methyl ester (28.87%), and 9-Octadecenoic acid (Z)-, methyl ester (16.58%). *J. rubens* (ethyl acetate extract) contained compounds such as Phytol (24.65%) and 9,12,15-Octadecatrienoic acid, methyl ester, (Z, Z, Z)- (20.03%), along with Hexadecanoic acid, methyl ester (15.48%). *C. racemose* (methanol extract) was rich in compounds like spathulenol (1.29%), Cubenol (0.45%), 2-Cyclohexen-1-one, 2-methyl-5-(1-methyl phenyl) (0.41%), and trans-calamine (0.3%) as summarized in Table 5 and Fig. 8.

**Table 5:** Major bioactive chemical compounds identified in the most effective algal extracts.

Algae	Extract	S. No.	RT (min.)	Compound Name	Molecular formula	Molecular weight	Peak area (%)
*Halimeda opuntia*	Acetone	1	16.83	Tetradecanoic acid, methyl ester	C_15_H_30_O_2_	242	0.14
		2	18.79	Tetradecanoic acid, 12-methyl-, methyl ester	C_16_H_32_O_2_	256	0.05
		3	19.14	2-Pentadecanone, 6,10,14-trimethyl-	C_18_H_36_O	268	0.08
		4	20.85	Hexadecanoic acid, methyl ester	C_17_H_34_O_2_	270	28.87
		5	22.01	n-Hexadecanoic acid	C_16_H_32_O_2_	256	2.66
		6	23.95	9,12-Octadecadienoic acid (Z,Z)-, methyl ester	C_19_H_34_O_2_	294	42.35
		7	24.05	9-Octadecenoic acid (Z)-, methyl ester	C_19_H_36_O_2_	296	16.58
		8	24.44	Octadecanoic acid, methyl ester	C_19_H_38_O_2_	298	4.71
		9	24.98	9,12-Octadecadienoic acid (Z,Z)-	C_18_H_32_O_2_	280	4.00
		10	27.22	Hexadecadienoic acid, methyl ester	C_17_H_30_O_2_	266	0.11
		11	27.78	13,16-Octadecadiynoic acid, methyl ester	C_19_H_30_O_2_	290	0.07
		12	31.17	Diisooctyl phthalate	C_24_H_38_O_4_	390	0.39
*Jania rubens*	Ethyl acetate	1	10.32	4-Hydroxy-4-methyl-4H-naphthalen-1-one	C_11_H_10_O_2_	174	0.40
		2	11.05	2-Propenoic acid, 3-phenyl-, methyl ester	C_10_H_10_O_2_	162	1.45
		3	13.97	11,13-Dihydroxy-tetradec-5-ynoic acid, methyl ester	C_15_H_26_O_4_	270	0.37
		4	14.26	( +) spathulenol	C_15_H_24_O	220	1.20
		5	14.49	4-epi-cubedol	C_15_H_26_O	222	0.77
		6	15.29	tau-Cadinol	C_15_H_26_O	222	3.06
		7	15.43	Arachidonic acid methyl ester	C_21_H_34_O_2_	318	0.39
		8	16.81	13,16-Octadecadiynoic acid, methyl ester	C_19_H_30_O_2_	290	0.53
		9	18.95	Neophytadiene	C_20_H_38_	278	1.87
		10	19.13	2-Pentadecanone, 6,10,14-trimethyl-	C_18_H_36_O	268	1.77
		11	19.77	17-Octadecynoic acid	C_18_H_32_O_2_	280	1.83
		12	20.74	Hexadecanoic acid, methyl ester	C_17_H_34_O_2_	270	15.48
		13	21.97	n-Hexadecanoic acid	C_16_H_32_O_2_	256	7.62
		14	23.07	Cholestan-3-ol, 2-methylene-,(3á,5à)-	C_28_H_48_O	400	0.59
		15	23.24	2,2,3,3,4,4 Hexadeutero Octadecanal	C_18_H_30_D_6_O	268	0.28
		16	23.76	9,12-Octadecadienoic acid (Z,Z)-, methyl ester	C_19_H_34_O_2_	294	3.58
		17	23.92	9,12,15-Octadecatrienoic acid, methyl ester, (Z,Z,Z)-	C_19_H_32_O_2_	292	20.03
		18	24.18	Phytol	C_20_H_40_O	296	24.65
		19	24.38	Octadecanoic acid, methyl ester	C_19_H_38_O_2_	298	1.32
		20	25.34	Oleic Acid	C_18_H_34_O_2_	282	1.16
		21	27.73	2-Propenoic acid, 3-phenyl-, methyl ester	C_10_H_10_O_2_	162	5.72
		22	28.24	Torreyol	C_15_H_26_O	222	0.17
		23	29.19	Isochiapin B	C_19_H_22_O_6_	346	4.47
		24	31.13	Diisooctyl phthalate	C_24_H_38_O_4_	390	1.30
*Caulerpa racemosa*	Methanol	1	7.59	Cyclohexanol, 2-methyl-5-(1-methylethenyl)-	C_10_H_18_O	154	0.08
		2	8.45	2-Cyclohexen-1-one, 2-methyl-5-(1-methylethenyl)-	C_10_H_14_O	150	0.41
		3	12.25	á-copaene	C_15_H_24_	204	0.19
		4	13.07	Cyclohexan-1-ol-2-carboxylic acid, 2-allyl-3-methyl-, methyl ester (1R,2S)-	C_12_H_20_O_3_	212	0.14
		5	13.29	trans-calamenene	C_15_H_22_	202	0.3
		6	13.37	Phenol, 2,4-bis(1,1-dimethylethyl)-	C_14_H_22_O	206	0.09
		7	14.26	spathulenol	C_15_H_24_O	220	1.29
		8	14.35	Caryophyllene oxide	C_15_H_24_O	220	0.19
		9	14.84	Cubenol	C_15_H_26_O	222	0.45
		10	15.50	à-Cadinol	C_15_H_26_O	222	0.27
		11	16.08	2,5-Octadecadiynoic acid, methyl ester	C_19_H_30_O_2_	290	0.15
		12	16.82	13,16-Octadecadiynoic acid, methyl ester	C_19_H_30_O_2_	290	0.96
		13	19.16	2-Pentadecanone, 6,10,14-trimethyl-	C_18_H_36_O	268	6.82
		14	19.99	Hexadecanoic acid, methyl ester	C_17_H_34_O_2_	270	0.46
		15	20.27	9-Hexadecenoic acid	C_16_H_30_O_2_	254	0.31
		16	20.85	Hexadecanoic acid, methyl ester	C_17_H_34_O_2_	270	31.51
		17	22.03	n-Hexadecanoic acid	C_16_H_32_O_2_	256	5.69
		18	23.79	9,12-Octadecadienoic acid, methyl ester	C_19_H_34_O_2_	294	6.18
		19	23.98	9,12,15-Octadecatrienoic acid, methyl ester, (Z,Z,Z)-	C_19_H_32_O_2_	292	22.1
		20	24.16	Phytol	C_20_H_40_O	296	6.91
		21	24.40	Octadecanoic acid, methyl ester	C_19_H_38_O_2_	298	4.95
		22	25.00	9,12,15-Octadecatrienoic acid, (Z,Z,Z)-	C_18_H_30_O_2_	278	1.23
		23	25.93	Hexadecadienoic acid, methyl ester	C_17_H_30_O_2_	266	0.76
		24	26.71	Oleic Acid	C_18_H_34_O_2_	282	3.73
		25	27.18	9,12-Octadecadienal, dimethyl acetal	C_20_H_38_O_2_	310	1.36
		26	27.75	Eicosanoic acid, methyl ester	C_21_H_42_O_2_	326	1.03
		27	27.85	10-Heptadecen-8-ynoic acid,methylester, (E)-	C_18_H_30_O_2_	278	0.83
		28	29.18	Isoaromadendrene epoxide	C_15_H_24_O	220	0.1
		29	30.87	Docosanoic acid, methyl ester	C_23_H_46_O_2_	354	0.28
		30	31.13	Diisooctyl phthalate	C_24_H_38_O_4_	390	0.46
		31	31.56	17-Octadecynoic acid	C_18_H_32_O_2_	280	0.35
		32	34.27	1-Heptatriacotanol	C_37_H_76_O	536	0.12
		33	42.98	á-Sitosterol	C_29_H_50_O	414	0.32

### Real-time PCR analysis

**Fig. 9:**
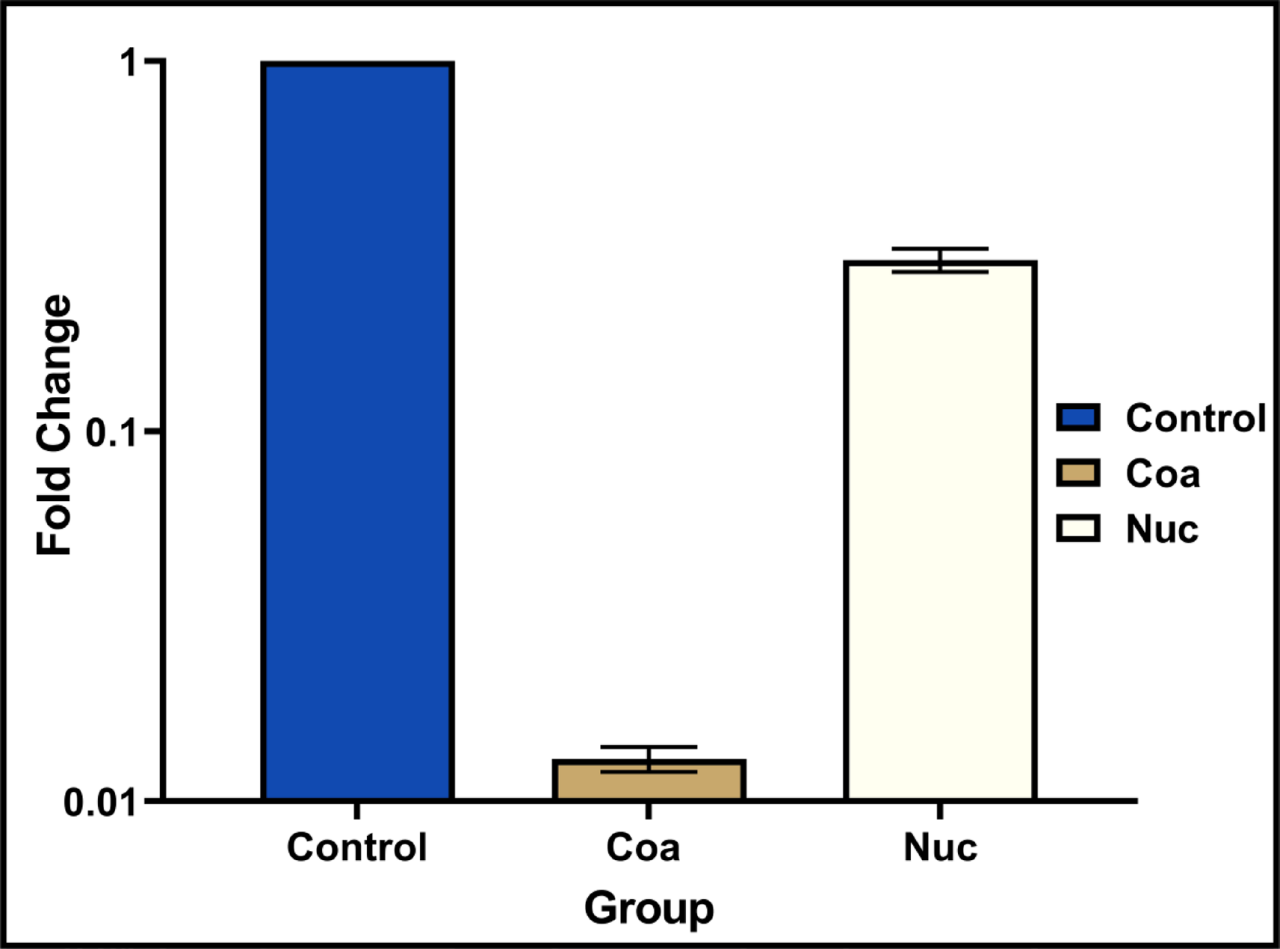
RT-PCR analysis of virulence gene expression (*nuc* and *coa*) in response to (*C. racemosa*) algal extract treatment (n = 10).

The RT-PCR analysis revealed significant downregulation of virulence genes in seaweed extract-treated *S. aureus* compared to untreated controls. The *coa* gene showed particularly strong suppression (98.7% reduction, fold change = 0.013), in addition to the *nuc* gene, which was affected (68.8% reduction, fold change = 0.312). The housekeeping gene (*16S rRNA*) demonstrated only minor CT value differences between groups (ΔCT = 2.15), suggesting the treatment specifically targets virulence factors rather than causing generalized bacterial toxicity. Amplification curves showed clear separation between control and treated samples, with later CT values in treated groups consistent with reduced target gene expression as shown in Table 6, Fig. 9, and Supplementary Fig. S2.

**Table 6:** Genes down regulation in *S. aureus*, detected by RT-PCR.

Group	*Housekeeping gene: 16S rRNA*	*coa*		*nuc*	
	CT	CT	Fold change	CT	Fold change
Control	8.5	14.40	1	24.64	1
Treated	10.65	22.80	0.013 ± 0.001***	27.87	0.312 ± 0.03**

*p* value (**) is less than 0.01, and (***) is less than 0.001.

## Discussion

Ready-to-eat (RTE) food is becoming increasingly popular among restaurants and street sellers worldwide, including in Egypt. Although RTE products offer the convenience of fast food, they also represent a risk of bacterial infection as they are not heated further. *S. aureus* cross-contamination of meat products is a leading cause of food poisoning globally^37^. The current research explored the ubiquitous distribution of *S. aureus* in the analyzed RTE beef products. In the current study, 30.7% of RTE-tested samples were positive for *S. aureus*. Similar findings were reported previously in Shaanxi Province, China (34.4%)^37^ and Libya (32%)^38^. In comparison, the obtained results are higher than those documented by other foodborne illness studies on RTE beef products that were 11.8% in China^39^ and 23.4% in Zagazig city, Egypt^40^. Otherwise, this result was lower than stated in beef products at Qalubeya Governorate, Egypt (50.8%)^41^.

Certain restaurant staff members need to gain awareness of and adhere to hygienic protocols, particularly personal hygiene-related ones; others may partially or fully observe these protocols. The fact is that the majority of workers don’t wear aprons, gloves, or protective head covers. Rather, some employees eat, drink, and even smoke while they work. According to recent studies, luncheon samples eaten immediately without being heated had lower *S. aureus* levels than those (kofta, burger, and shawarma) previously^39^. In contrast, our results highlighted that the highest incidence of *S. aureus* among RTE beef products was in luncheon samples by 36.7% with a mean of 6.1 × 10 ± 0.4 × 10 CFU/g. This confirms inadequate sanitary practices at the time of luncheon preparation and processing.

Moreover, Food handlers may be responsible for contaminated meat with *S. aureus* due to cross-contamination from their hands^42^. Our results agreed well with those of other studies in Egypt^41,43^. Furthermore, Morshdy et al.^43^ declared that *S. aureus* was more prevalent in Egypt’s kofta (90%), luncheon (50%), burger (75%), and shawarma (80%). These variations in meat microbiological quality can be attributed to factors such as cooking method (frying, roasting, or grilling), raw ingredient quality, meat size and shape, cooking utensils (stew container, grill, oven), and seasoning (dressings, vegetables, herbs, spices)^44^.

The high frequency of *S. aureus* in beef products may be because certain employees follow hygiene procedures inside these restaurants, wiping down contact surfaces that are exposed to food, like tables, preparation boards, and utensils, using just a towel or some soap and water. On rare occasions, some continue to work while experiencing hand or respiratory problems like boils or coughing. Humans experience a wide range of diseases as a result of these conditions. Therefore, enhancing processing methods, monthly checks of workers and utensils for *S. aureus*, frequent disinfection of food contact surfaces, proper heating of beef products, and finally, strict personnel hygiene measures are all important to prevent the existence of *S. aureus* in RTE beef products^42^.

The current study declared that 58.7% of the *S. aureus* isolates from the samples that were looked at had coagulase, 47.6% had *nuc*, and 13% had only *seb* amongst other S.E.s encoding genes. More than 13% of *S. aureus* confined from RTE beef products were affirmative for more than one virulence-associated gene (Table 2). Amplification of the coagulase gene proved to be a rapid and precise approach for typing *S. aureus*. All explored isolates of *S. aureus* produce the coagulase gene, a highly virulent component. Coagulase results in plasma coagulation in the host and is an identifying marker for *S. aureus* infection^45^. The existence of *coagulase* and *nuc* genes explored in this study is comparable with previous studies ^45,46^.

Additionally, S.E.s are the most prominent virulence genes in *S. aureus* and the leading cause of staphylococcal food poisoning. The weak evidence of staphylococcal food poisoning outbreaks with the classical enterotoxins *seb, sec, sed,* and *she* can be identified commercially^47^. Although the Egyptian Organization for Specification and Quality Control^48^ requires that S.E.s not be detected in beef products, our study detected the *seb* gene in 13% of isolated *S. aureus*, which produces enterotoxins. In addition, Sallam et al. ^49^ identified *sea, seb,* and *sec* genes in all positive isolates. Wu et al.^5^ concluded that all *S. aureus* isolates had a minimum of one enterotoxin gene.

The incidence of S.E. genes in a wide range of isolates underlines their vertical transmission potential rather than horizontal transmission, as most of these genes exist in mobile parts of the genome. *S. aureus* enterotoxin explored gene differs by country due to geographical variations and is further influenced by the isolated strains’ ecological sources (food, animals, and humans). Moreover, in Poland, *sec*; Bulgaria, *sea*; and Egypt, *seb* are the most common toxin genes ^46,50,51^. The isolates’ toxin gene profiles vary according to their origin, isolation samples, and geographical location.

On the other hand, resistance to penicillin, cefoxitin, and oxacillin was significantly detected in the *S. aureus* isolates in this study. This finding aligns with Wu et al.^5^ and Abbasi et al.^52^, who documented an increase in β-Lactam resistance among *S. aureus* isolates. Also, strong resistance to aminoglycoside antibiotics was reported in previous studies, except Abbasi et al.^52^, who reported lower resistance to kanamycin, tetracycline, and Chloramphenicol. Moreover, Komodromos et al.^53^ reported similar penicillin resistance and lower Sulfamethoxazole/Trimethoprim, ampicillin, cefoxitin, tetracycline, and chloramphenicol resistance. The study found that 50% of the isolates resisted cefoxitin and were considered MRSA. This study has also revealed that shawarma had the highest occurrence of MRSA isolates, accounting for 55.5% of the samples. Following closely behind were Kofta, burger, and Sausage, each with a frequency of 50%. In contrast to the global meta-analysis, which reported a detection rate of 3.2% for MRSA in beef products, the current study reveals a significantly higher prevalence of this bacterium^54^. The rate of MRSA isolation observed in this study also exceeds that reported by Mahros et al.^42^ and Song et al. ^55^. However, the frequency was lower than reported by Saber et al.^40^.

In the current study, 15 out of 23 MRSA investigated were positive for the *mec*A gene, and 8 out of 23 MRSA did not show any presence of the *mec*A gene. These alternative *mec* genes, such as *mec*B or *mec*C, could be responsible for their methicillin resistance. According to Yang et al.^39^ and Abbasi et al.^52^, the prevalence of *mec*A was found in 6 and 32 MRSA strains, while Saber et al. ^40^ and Al-Amery et al. ^56^ amplified *mec*A in all MRSA strains. Furthermore, Vancomycin is the optimal therapeutic option for treating methicillin-resistant biofilm infections^57^. However, the World Health Organization has categorized vancomycin-resistant *S. aureus* (VRSA) as a “high priority” antibiotic-resistant pathogen due to its significant impact on public health. The percentage of VRSA found in our samples was higher than that found by Yang et al.^39^ and Al-Amery et al.^56^ in previously published studies. Notably, none of the isolates obtained by Wang et al.^37^ and Abbasi et al.^52^ exhibited vancomycin resistance. The varying levels of antimicrobial resistance observed across various studies may be attributed to the differences in opinions among veterinary physicians about which type of antimicrobial agents should be prescribed, the practicality of regulations that limit their use, and the pricing of such agents. These factors can significantly impact the prevalence of antimicrobial resistance within distinct geographical regions.

Eleven distinct antimicrobial resistance profiles and 39 MDR isolates were revealed. The MDR isolates observed in our study exhibit higher levels when compared to the findings reported by Velasco et al.^58^, and Ou et al.^59^. Such variations in resistance profiles can have significant implications for the treatment and control of infections caused by this bacterium. The antimicrobial susceptibility evaluation has yielded results indicating a notable occurrence of resistance towards specific antimicrobials, with a range of resistance patterns identified among the isolates of *S. aureus*^60^. This can be attributed primarily to the extensive and inconsistent utilization of these agents, particularly in veterinary medicine, resulting in a significant increase in the prevalence of antimicrobial resistance. Moreover, the excessive application of disinfectants, self-treatment with antimicrobial agents, and the provision of single-dose therapies have all contributed to this issue. Hence, it is crucial to promptly adopt rigorous preventive strategies to restrict the transmission of antimicrobial resistance across all stages of food production^60^.

Biofilm formation was a common characteristic among most strains of *S. aureus*. Our findings are consistent with previous research, highlighting the ability of *S. aureus* isolates from meat and meat products to form biofilms^52,53^. The production of biofilms by *S. aureus* and their simultaneous presence with saprophytic microorganisms within these biofilms undeniably gives rise to the establishment of persistent contamination reservoirs within food-processing facilities^44^. These findings are supported by Ou et al.^59^, as they revealed that food serves as an excellent adhesive medium and a reservoir for *S. aureus* because the common properties among food substrates, particularly their viscosity, play an overwhelmingly significant role in the successful colonization of *S. aureus* as compared to the differences in food surface properties and bacterial species.

The role of the *ica* genes in forming biofilms by *S. aureus* strains has been extensively explored. It has been consistently observed that the majority of biofilm-producing *S. aureus* strains in the current study possess the *ica*A and *ica*D genes either separately or in combination, with the *ica*D being predominant, which disagrees with the study of Saber et al.^40^ where the *ica*A was the most prevalent demonstrates a significant correlation with the formation of biofilms. The results obtained in this study align with Tang et al.^61^, who found that 87.5% of *S. aureus* strains isolated from different food sources possessed the *ica*A and *ica*D genes. This indicates a consistent presence of these genes in *S. aureus* strains across various origins. Furthermore, Abbasi et al.^52^ detected *ica*A and *ica*D genes in biofilm-producing *S. aureus* obtained from raw meat and meat products (80.4% each). These findings emphasize the importance of the *ica*A and *ica*D genes in forming biofilms by *S. aureus*, which can affect food safety.

In this study, isolates containing the *ica*D gene produced biofilms that ranged from moderate to strong. In contrast, isolates that expressed both *ica*A and *ica*D exhibited strong biofilm (*p* < 0.05). Mubarak and El-Zamkan^62^ confirmed these results. Contrarily, it has been ascertained that three isolates were identified to have solely the *ica*A gene and were linked with a weak biofilm phenotype. A study conducted by Gerke et al.^63^ explained the link between genotype and phenotype. They found that *ica*A alone had a weak N-acetylglucosaminyl transferase activity, but when *ica*D was co-transcribed with *ica*A, it had full activity. Biofilm is one of the useful tools used by *S. aureus* to resist antibiotics^59^, which illustrates the significant relationship between biofilm formation and MDR in this study.

The study’s findings are quite alarming as they reveal that a significant number of MRSA strains (60.9%) are also VRSA, a major cause of concern. Moreover, 21.7% of these strains have developed resistance against both vancomycin and Linezolid. These results highlight the need for urgent action to address the escalating concern of antibiotic resistance and to find new ways to combat these pathogens. Also, this study reveals that all MRSA are MDR and biofilm producers, except three isolates, non-biofilm producers, while 14 out of 18 VRSA (77.8%) are biofilm producers. *S. aureus* biofilm-associated infections give rise to severe and potentially fatal diseases, including endocarditis, septic arthritis, cystic fibrosis, and recurrent infections in both human and animal communities^64^. This finding underscores the significant impact of MRSA as an opportunistic pathogen in nosocomial infections, as it demonstrates high levels of resistance to various antibiotics and possesses a wide range of virulence factors. What adds to the concern is that a considerable proportion of MRSA strains (60.9%) also exhibit resistance to vancomycin, a major cause of concern. Moreover, 35.7% of MRSA and VRSA strains resist Linezolid, which is crucial in MRSA treatment. Another problem is that 4 MRSA/VRSA isolates harbored the *seb* gene. Identifying beef products as a reservoir for virulence and antibiotic-resistant *S. aureus* highlights the pressing need for prompt intervention. These findings emphasize the urgency of taking action to mitigate the issue of antibiotic resistance and to discover alternative methods for combating these organisms.

The 32.6% MRSA confirmation rate in ready-to-eat meat products represents a significant public health threat, particularly for vulnerable populations including hospitalized patients, immunocompromised individuals, and elderly consumers. The presence of virulent MRSA strains carrying both resistance (*mec*A) and virulence (*nuc*) genes in commonly consumed foods highlights the urgent need for integrated surveillance and control measures spanning the entire food-to-healthcare continuum^9,11^.

Protecting human health from the harmful consequences of hostile *S. aureus* has become increasingly challenging. The chemical preservatives’ destructive effects on human health, microbial resistance, toxicity, susceptibility, and their limited application have upsurged the demand for potentially effective, safer, healthy, and natural antimicrobial agents with a unique force against pathogens, especially *S. aureus*^12^. Thus, the antibacterial activity of algal extracts can provide a crucial component of *S. aureus* infection remedy that traditional antibacterial agents cannot. In addition, they can be used as organic preservatives to safeguard healthier and safer food away from the risks and harmful side effects of ordinary chemical preservatives^12^. In the current study, three algal species (crude extracts), including *H. opuntia*, *J. rubens,* and *C. racemosa,* exerted strong bactericidal activity, dose-dependently, against *S. aureus* according to seaweed species and used solvent. Persuasive antibacterial activity was recognized for *C. racemosa* in methanol extract against *S. aureus,* resulting in a reduction of serious bacterial growth (Δabs 620) at a 1.5 mg/ml concentration. However, the concentrations of 1.5 mg/ml of *J. rubens* (ethyl acetate extract) and *H. opuntia* (acetone extract) showed much less bacterial growth reduction (Δabs 620) in *S. aureus* (Fig. 7).

Diverse studies explored the antibacterial potential activities of marine seaweed, viz., *J. rubens*, *H. opuntia*, and *C. racemosa*. Different solvents, viz., petroleum ether, acetone, chloroform, methanol, ethyl acetate, ethanol, hexane, and water, were practiced for algae extraction to explore the antimicrobial algal extract activity against bacterial cultures, including *S. aureus*^12,65^. *C. racemosa* (methanol extract) exhibits the strongest activity against the growth of MRSA (methicillin-resistant *S. aureus*) with the highest antimicrobial effect (97.7 ± 0.30%)^58^. Similar results were obtained by Chan et al.^66^, using the broth microdilution technique, where methanolic, hexane, acetone, chloroform, ethyl acetate, and ethanolic extracts of *C. racemosa* showed different activity against Gram-positive bacteria growth (*S. aureus*) with a 0.36 mm inhibition zone.

Furthermore, the *C. racemosa* extract exhibits a dual mechanism of action against *S. aureus*, combining direct bactericidal effects with targeted virulence suppression. The profound inhibition of *nuc* and *coa* genes detected by RT-PCR analysis suggests the extract disrupts both immune evasion (via nuclease suppression) and clotting mechanisms (via coagulase reduction). While the log reduction demonstrates killing capacity, the virulence gene suppression occurs even at sub-lethal concentrations, potentially reducing selective pressure for resistance. This combined approach—simultaneously decreasing pathogen load and disabling virulence factors—may prove more effective than conventional antibiotics. Future studies should identify the active compounds responsible for these effects and evaluate their efficacy in animal infection models.

Moreover, it was reported that *C. racemosa* exhibited promising antimicrobial activity against human and food pathogens^67,68^. The promising efficacy of the marine seaweed *C. racemosa* extract against the *S. aureus* pathogen could be due to the active metabolites, phytochemicals, and fatty acids as well as their derivatives^65,66^. The promising antibacterial effects of methanol extract of C. racemosa against *S. aureus* could be peculiarities to the most bountiful detected phytochemicals and bioactive compounds including spathulenol (1.29%), Cubenol (0.45%), 2-Cyclohexen-1-one, 2-methyl-5-(1-methylethenyl) (0.41%), trans-calamenene (0.3%).

Additionally, in vitro data in previous literature showed that Spathulenol poses potent antimicrobial activities against diverse bacterial cultures, including *S. aureus*^67^. It has been declared that spathulenol has a major role in anti-inflammatory, immunomodulatory, and antiproliferative activities, with non-toxic, non-mutagenic, and non-tumorigenic properties, and could be a potential antimicrobial drug^69^. Moreover, Cubenol and 2-Cyclohexen showed antibacterial activities against *S. aureus* with MICs ranging from 7.81  to 15.62 mg/ml. Moreover, a promising antifungal activity against filamentous fungi was detected, which could be utilized in wide-ranging applications to prevent microbial growth as drug molecules, cosmetics, and food ^70^.

It is reasonable to assume that the detected compounds have potential antibacterial features and that extracts of the algae in which they are abundant have powerful antibiotic properties. The investigated algae species may be more efficient against pathogenic bacteria than typical bactericidal treatments. Hence, they may be regarded as natural preservatives, delivering nutritious and safe food through their stable, biologically active molecules without the adverse effects of chemicals. It also offers perspectives on discovering innovative antibacterial agents for food preservation or therapeutic applications.

The observed antimicrobial activity of the algal extracts can be attributed to the presence of bioactive constituents, which have been previously reported for their antibacterial properties. For instance, spathulenol has demonstrated significant antimicrobial effects^71^, while cubenol has been shown to inhibit bacterial growth^72^. Similarly, 2-cyclohexen-1-one, 2-methyl-5-(1-methylethenyl)^73^ and trans-calamenene^74^ have also been documented for their antibacterial activities. These compounds likely contribute to the overall antimicrobial efficacy of the algal extracts by targeting bacterial cell membranes, disrupting enzymatic functions, or inducing oxidative stress. The presence of such bioactive constituents in the algal extracts underscores their potential as natural antimicrobial agents.

## Conclusion

The results of the current study indicate that significant samples under investigation are contaminated with MRSA and VRSA biofilm-producing *S. aureus*, which possess one or more virulence genes, increasing the risk of foodborne disease and potentially facilitating its transmission. Additionally, the study investigated the potential of Algal extracts as natural antimicrobial alternatives that benefit the reduction of *S. aureus* growth. This is a promising opportunity to create novel antibacterial compounds with enormous potential for application in food preservation and medicine. It is recommended that strict hygiene rules be consistently enforced in the following areas: food handling, food contact surfaces, personal sanitary practices, and the consumption of completely cooked beef products. To enhance future research, we recommend incorporating quantitative real-time PCR (qRT-PCR) for the precise downregulation assessment of target genes. Additionally, in-vivo experiments using murine models should be conducted to further validate the antimicrobial efficacy of algae. Additional research is required to identify other virulence and antibiotic-resistance genes in *S. aureus* and investigate other natural antimicrobials to achieve the best circumstances for extending the product’s shelf life. Our finding underscores the importance of implementing strict food safety protocols, enhanced antimicrobial stewardship programs, and coordinated One Health surveillance systems to prevent the transmission of hospital-grade pathogens through the food chain into clinical settings.

## Supplementary Information

Below is the link to the electronic supplementary material.

Supplementary Material 1

Supplementary Material 2

## Data Availability

All data supporting the findings of this study are available within the paper and its Supplementary Information. Microsatellite primer sequences are provided in Supplementary Tables 1 and 2, along with original references describing the microsatellites used in this study.
