# Supplementary material for: Unleashing the biological potential of marine algal extracts against Staphylococcus aureus isolated from ready-to-eat beef products
Source: Sci Rep. 2025 Aug 17;15:30111. doi: 10.1038/s41598-025-14674-w (PMC12358557; doi:10.1038/s41598-025-14674-w)
Supplement: Supplementary file 1 — Supplementary Material 1 [file 41598_2025_14674_MOESM1_ESM.docx]

**Supplementary Tables and Figures**

**Supplementary Table S1:** PCR protocol including primer sequences, Amplicon size and amplification reactions for *Staphylococcus aureus* virulence genes.

| Target gene | Primers sequences (5`-3`) | Amplified segment (bp) | Primary  denaturation | Amplification | | | Final extension | Reference |
| --- | --- | --- | --- | --- | --- | --- | --- | --- |
|  |  |  |  | Secondary denaturation | Annealing | Extension |  |  |
| *Sea* | GGTTATCAATGTGCGGGTGG | 102 | 94 ^˚^C  5 min. | (35 cycles) | | | 72 ^˚^C  10 min. | Mehrotra *et al.* (2000) ^22^ |
|  |  |  |  | 94 ^˚^C  30 sec. | 57 ^˚^C  40 sec. | 72 ^˚^C  40 sec. |  |  |
|  | GGTTATCAATGTGCGGGTGG |  |  |  |  |  |  |  |
| *Seb* | GTATGGTGGTGTAACTGAGC | 164 |  |  |  |  |  |  |
|  | CCAAATAGTGACGAGTTAGG |  |  |  |  |  |  |  |
| *Sec* | AGATGAAGTAGTTGATGTGTATGG | 451 |  |  |  |  |  |  |
|  | CACACTTTTAGAATCAACCG |  |  |  |  |  |  |  |
| *mecA* | GTA GAA ATG ACT GAA CGT CCG ATA A | 310 | 94 ^˚^C  5 min. | 94 ^˚^C  30 sec. | 50 ^˚^C  30 sec. | 72 ^˚^C  30 sec. | 72 ^˚^C  10 min. | McClure *et al.* (2006) ^23^ |
|  | CCA ATT CCA CAT TGT TTC GGT CTA A |  |  |  |  |  |  |  |
| *Coagulase* | ATA GAG ATG CTG GTA CAG G | Four different types of bands  350  430  570  630 | 94 ^˚^C  5 min. | 94 ^˚^C  30 sec. | 55 ^˚^C  40 sec. | 72 ^˚^C  45 sec. | 72 ^˚^C  10 min. | Lyer and Kumosani (2011)^24^ |
|  | GCT TCC GAT TGT TCG ATG C |  |  |  |  |  |  |  |
| *nuc* | ATATGTATGGCAATCGTTTCAAT | 395 | 94 ^˚^C  5 min. | 94 ^˚^C  30 sec. | 55 ^˚^C  40 sec. | 72 ^˚^C  40 sec. | 72 ^˚^C  10 min. | Gao *et al.* (2011) ^25^ |
|  | GTAAATGCACTTGCTTCAGGAC |  |  |  |  |  |  |  |
| *vanA* | GGGAAAACGACAATTGC | 732 | 94 ^˚^C  5 min. | 94 ^˚^C  30 sec. | 54 ^˚^C  45 sec. | 72 ^˚^C  45 sec. | 72 ^˚^C  10 min. | Depardieu *et al.* (2004) ^26^ |
|  | GTACAATGCGGCCGTTA |  |  |  |  |  |  |  |
| *optrA* | AGGTGGTCAGCGAACTAA | 1395 | 94 ^˚^C  5 min. | 94 ^˚^C  30 sec. | 53 ^˚^C  1 min. | 72 ^˚^C  1 min. | 72 ^˚^C  12 min. | Wang *et al.* (2015) ^27^ |
|  | ATCAACTGTTCCCATTCA |  |  |  |  |  |  |  |
| *icaA* | CCT AAC TAA CGA AAG GTA G | 1315 | 94 ^˚^C  5 min. | 94 ^˚^C  30 sec. | 49 ^˚^C  1 min. | 72 ^˚^C  1 min. | 72 ^˚^C  10 min. | Ciftci *et al.* (2009) ^28^ |
|  | AAG ATA TAG CGATAA GTG C |  |  |  |  |  |  |  |
| *icaD* | AAA CGTAAG AGA GGT GG | 381 | 94 ^˚^C  5 min. | 94 ^˚^C  30 sec. | 49 ^˚^C  30 sec. | 72 ^˚^C  30 sec. | 72 ^˚^C  7 min. |  |
|  | GGC AAT ATG ATC AAGATA |  |  |  |  |  |  |  |

**Supplementary Table S2:** Statistical analytical results of confirmed methicillin-resistant *S. aureus* (MRSA) in examined ready-to-eat meat products (N=30) according to antibiogram resistance pattern to cefoxitin, vancomycin, and linezolid; antibiotic resistance gene (*mec*A) and virulence gene (*Nuc*).

| Samples  (N = 30) | Positive samples  *S. aureus* | | Virulence gene | Antibiotic resistance gene | Antibiotic | | | No. (%) of samples positive for MRSA from *S. aureus* positive samples | |
| --- | --- | --- | --- | --- | --- | --- | --- | --- | --- |
|  | No. | % | *Nuc*  No. (%) | *mec*A  No. (%) | Cefoxitin No. (%) | Vancomycin No. (%) | Linezolid No. (%) | No. | % |
| Shawerma | 9 | 30 | 4 (44.4) | 3 (13) | 5 (55.6) | 3 (33.3) | 3 (33.3) | (3/9) | 33.3% |
| Kofta | 10 | 33.3 | 4 (40.0) | 3 (13) | 5 (50) | 5 (50) | 4 (40) | (3/10) | 30.0% |
| Burger | 8 | 26.7 | 5 (62.5) | 2 (8.7) | 4 (50) | 4 (50) | 0 (0) | (2/8) | 25.0% |
| Luncheon | 11 | 36.7 | 6 (54.5) | 4 (17.4) | 5 (45.5) | 3 (27.3) | 1 (9.1) | (4/11) | 36.4% |
| Sausage | 8 | 26.7 | 3 (37.5) | 3 (13) | 4 (50) | 3 (37.5) | 1 (12.5) | (3/8) | 37.5% |
| Total (150) | 46 | 30.67 | 22 (47.8) | 15 (65.2) | 23 (50) | 18 (39.1) | 9 (19.6) | (15/46) | 32.6% |

**Supplementary Table S3:** Antibacterial activity of algal extracts against *Staphylococcus aureus* at different concentrations. The data is presented as bacterial growth monitored at 620 nm. The assays were performed in triplicate.

| Concentration (µg/ml) [Algal extract based] | | Halimeda opuntia (Acetone) | Jania rubens (Ethyl acetate) | Caulerpa racemosa (Methanol) |
| --- | --- | --- | --- | --- |
| 0 | | 0.495 | 0.495 | 0.495 |
| 125 | | 0.485 | 0.46 | 0.472 |
| 250 | | 0.477 | 0.39 | 0.41 |
| 500 | | 0.461 | 0.361 | 0.35 |
| 1000 | | 0.449 | 0.35 | 0.3 |
| 1500 | | 0.425 | 0.335 | 0.275 |
| Norfloxacin | 10 μg/ml | 0.0 | | Positive control |
| Dimethyl sulfoxide (DMSO) | | 0.495 | | Negative control |

**Supplementary Table S4:** Killing power of algal extracts (1500, 1000, and 500 µg/ml) against *Staphylococcus aureus* as bacterial growth monitored at 620 nm. The assays were performed in triplicate.

| Concentration (µg/ml) [Algal extract based] | Halimeda opuntia (Acetone) | Jania rubens (Ethyl acetate) | Caulerpa racemosa (Methanol) |
| --- | --- | --- | --- |
| 1500 | 0.0662 | 0.1696 | 0.2553 |
| 1000 | 0.0424 | 0.1505 | 0.2175 |
| 500 | 0.0309 | 0.1371 | 0.1505 |

**
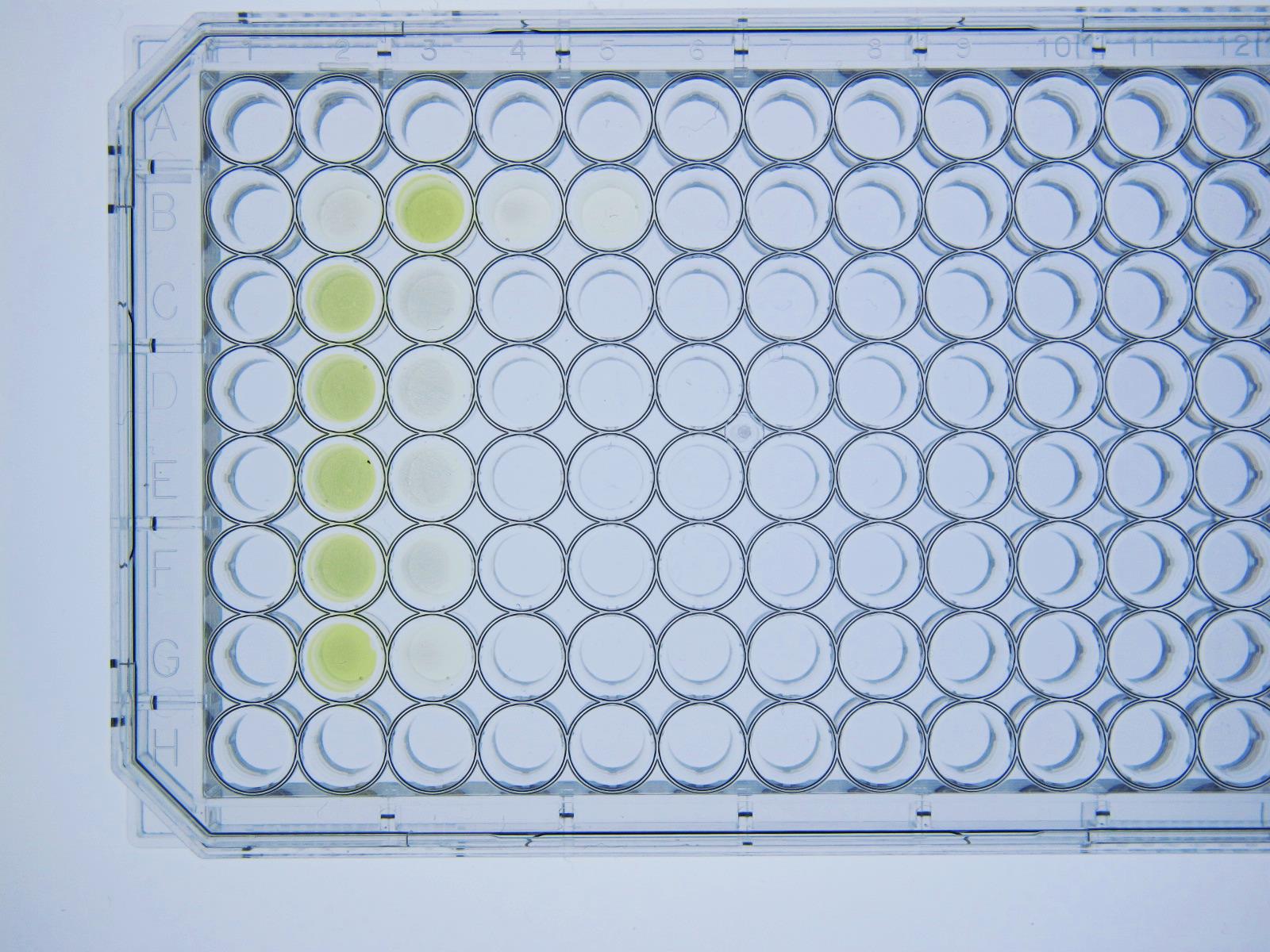
**

**Supplementary Figure S1:** Antibacterial activity of algal extracts against *Staphylococcus aureus* at different concentrations using 96-well microtiter plate. The data is presented as bacterial growth monitored at 620 nm.

**
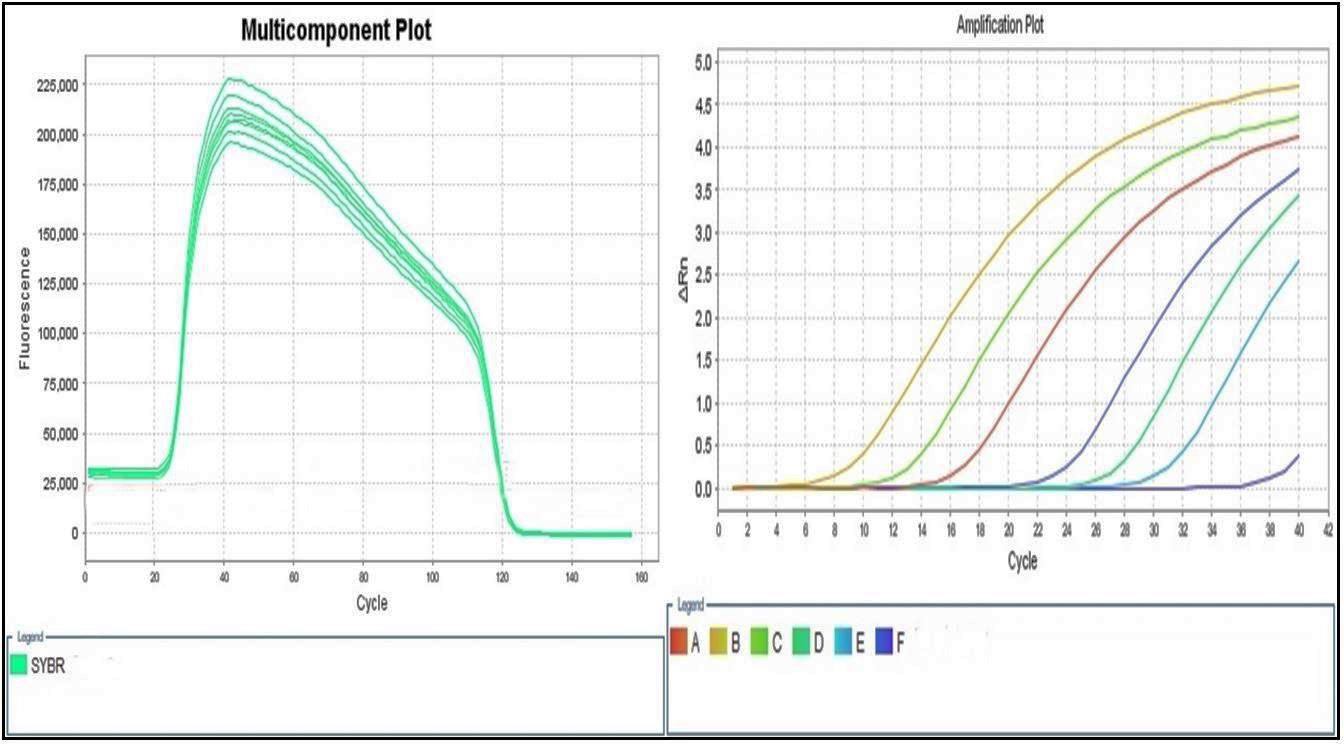
**

**Supplementary Figure S2:** RT-PCR (Multicomponent Plot and Amplification Plot)
