# Supplementary material for: Unleashing the biological potential of marine algal extracts against Staphylococcus aureus isolated from ready-to-eat beef products
Source: Sci Rep. 2025 Aug 17;15:30111. doi: 10.1038/s41598-025-14674-w (PMC12358557; doi:10.1038/s41598-025-14674-w)
Supplement: Supplementary file 2 — Supplementary Material 2 [file 41598_2025_14674_MOESM2_ESM.pdf]

630 bp for *coagulase* (*coa*) gene (A),

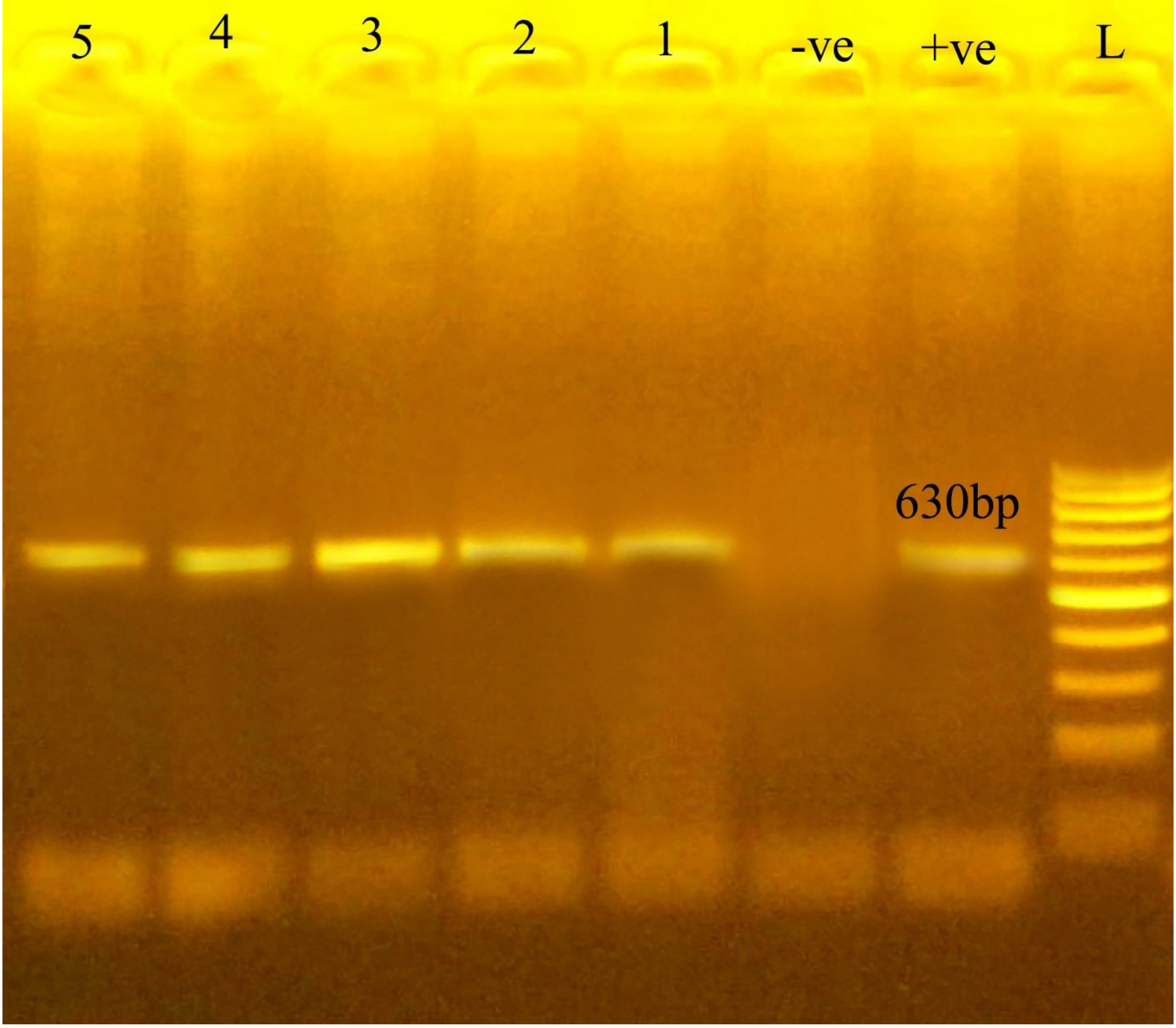

395 bp for *nuc* gene (B)

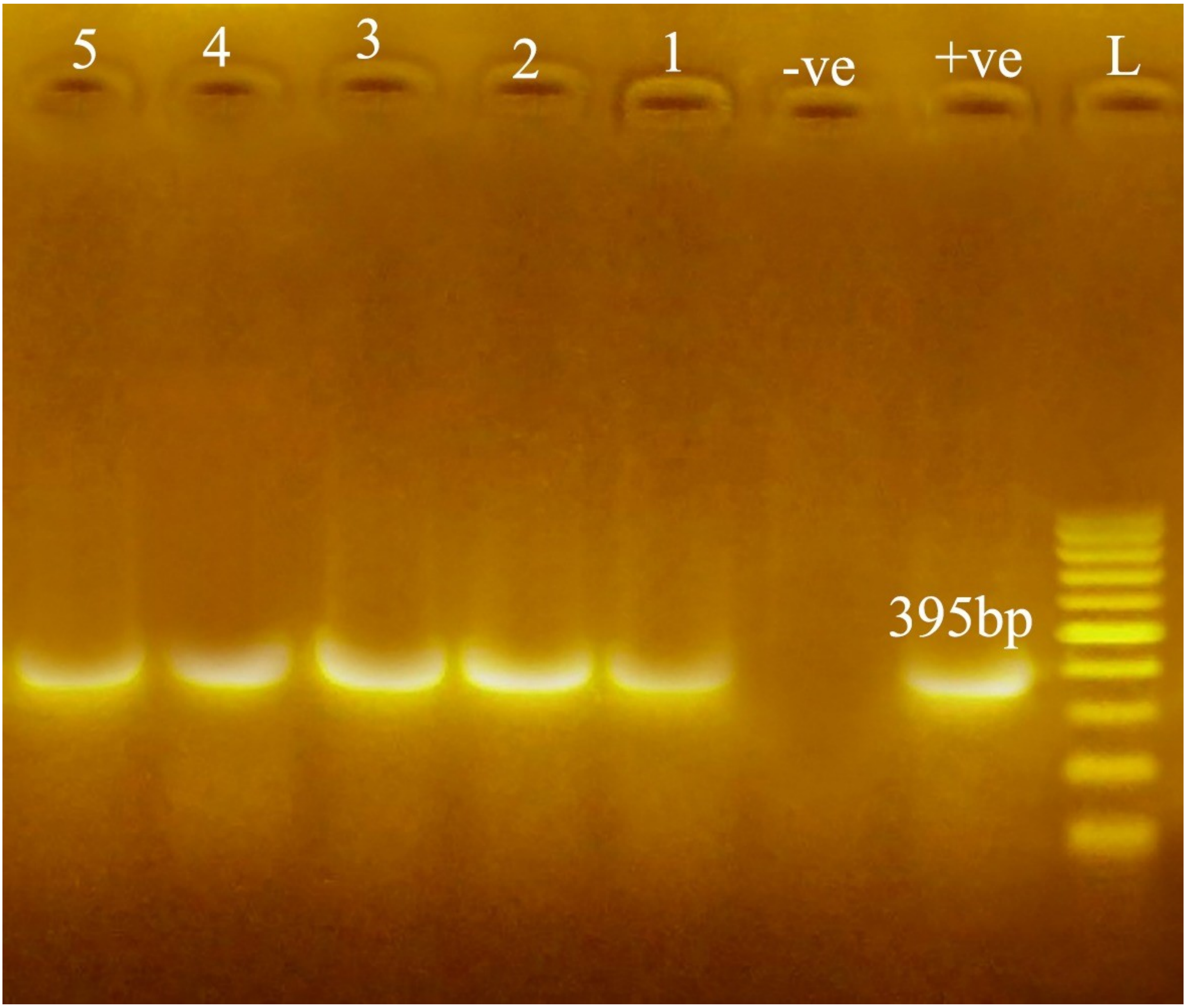

102 bp for *Sea* gene, 164 bp for *Seb* gene, 451 bp for *Sec* gene (C)

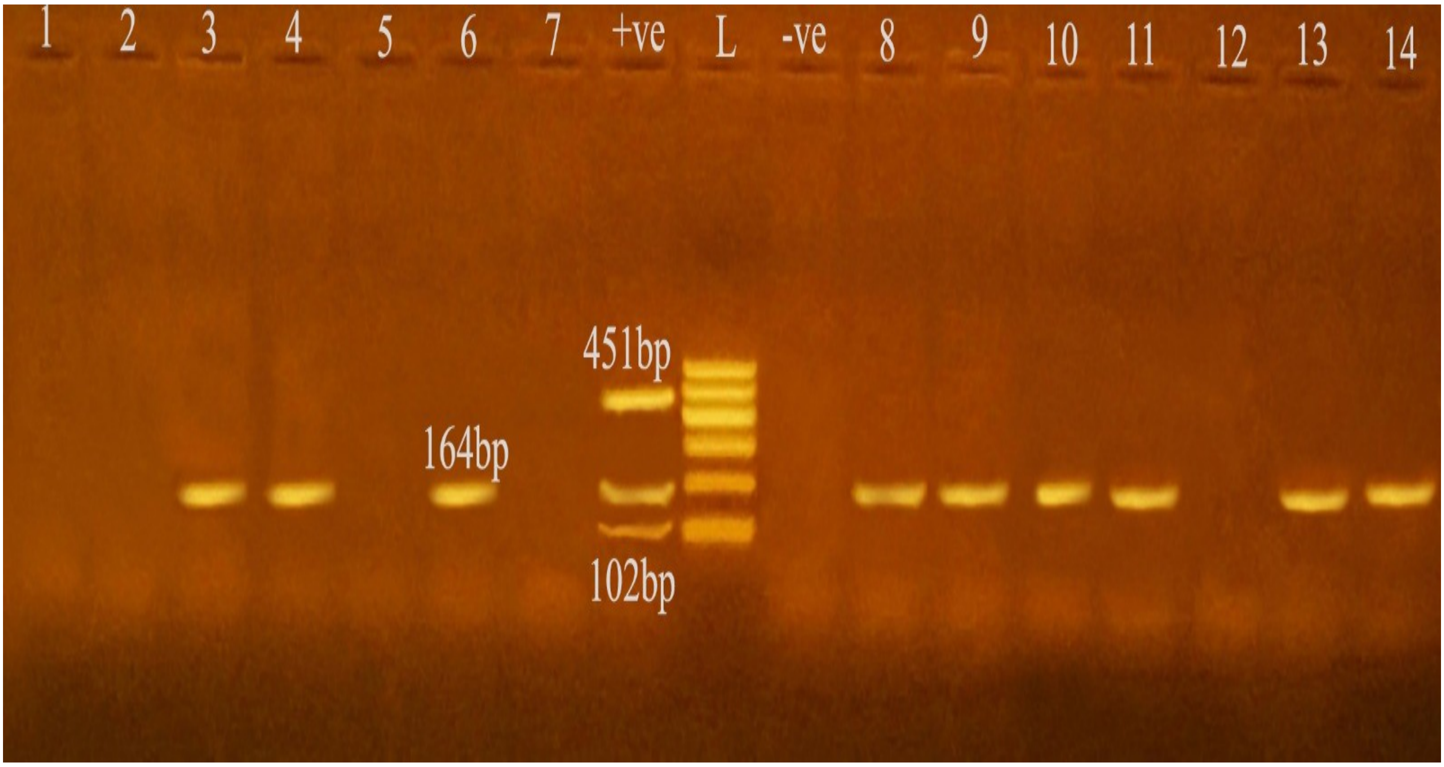

310 bp for *mec*A gene (D)

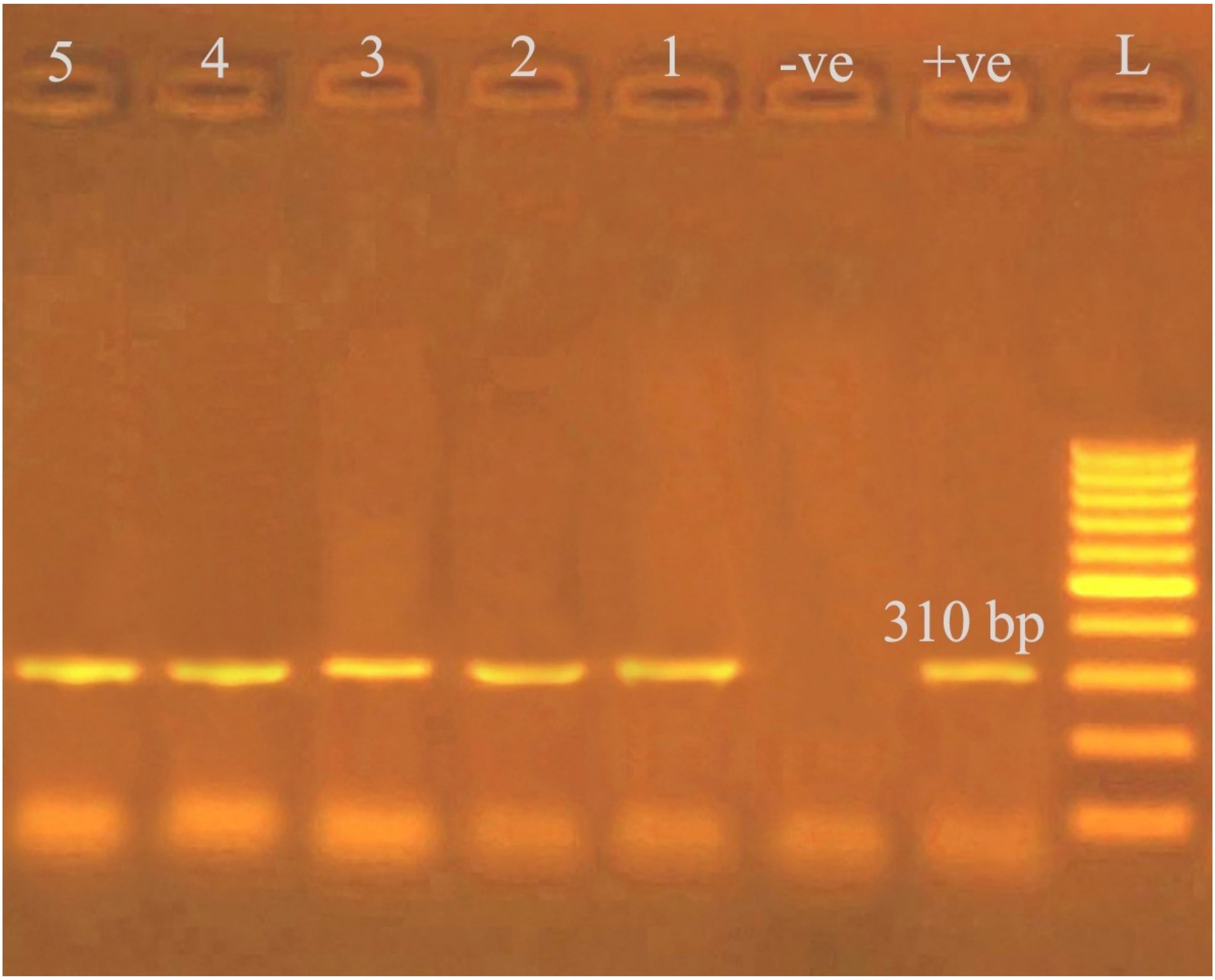

732 bp for *van*A gene (E)

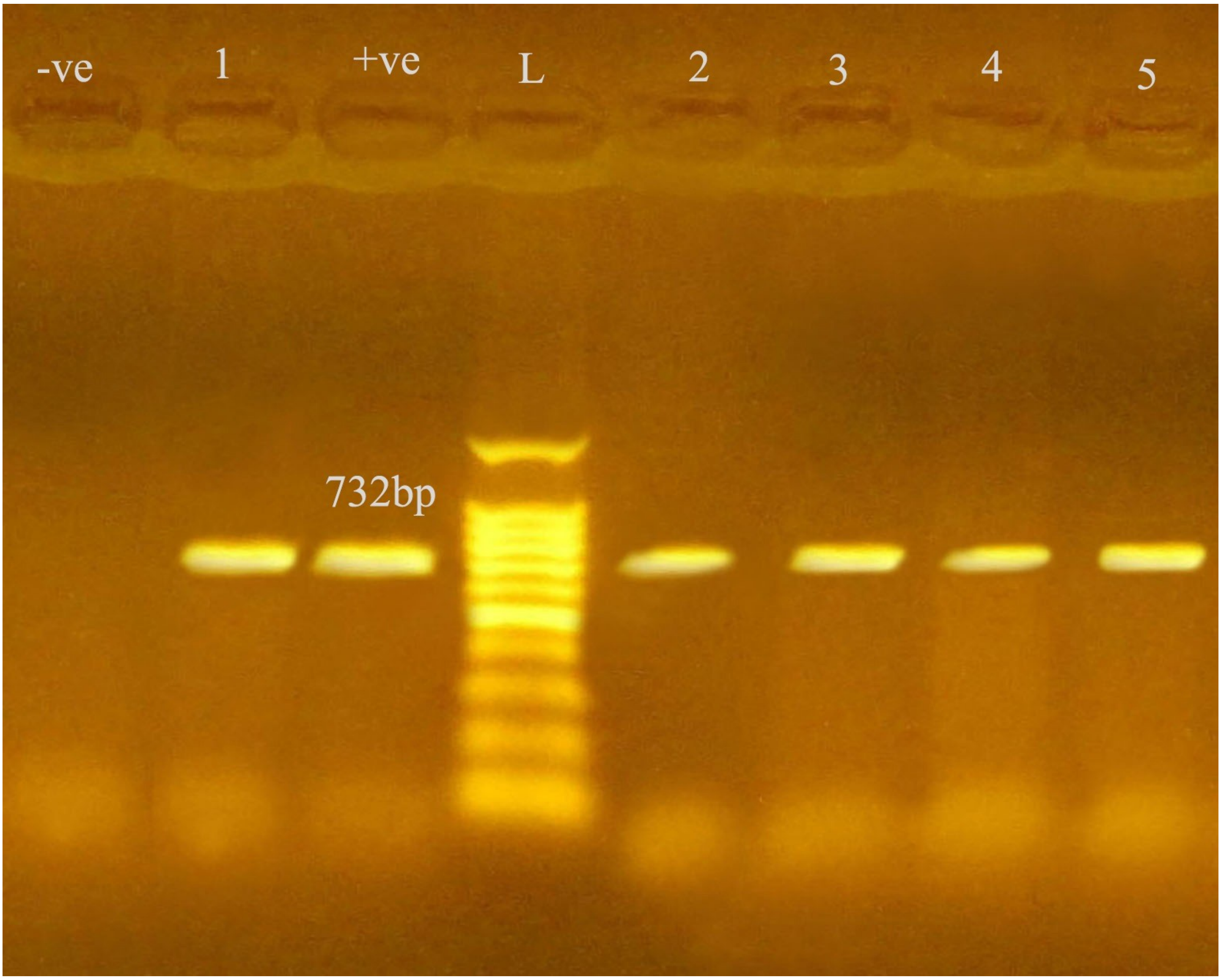

1395 bp for *optr*A gene (F)

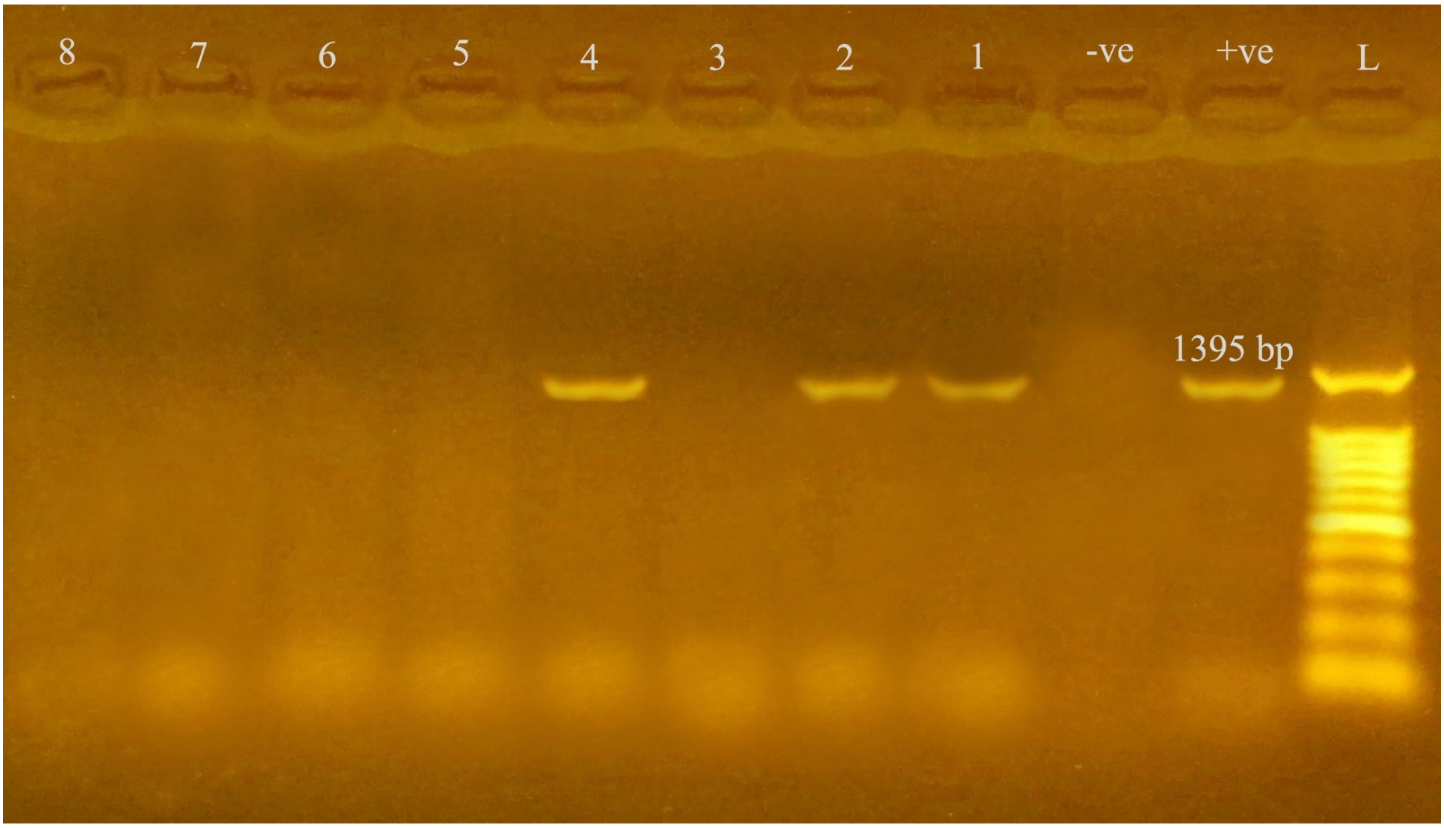

1315 bp for *ica*A gene (G)

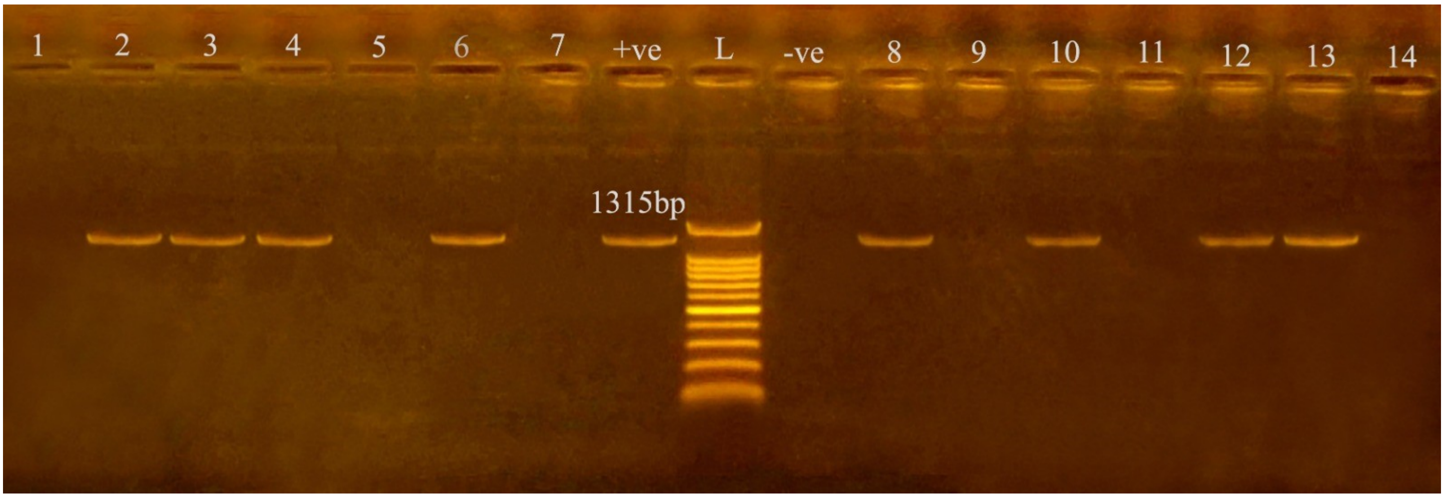

381 bp for *ica*D gene (H)

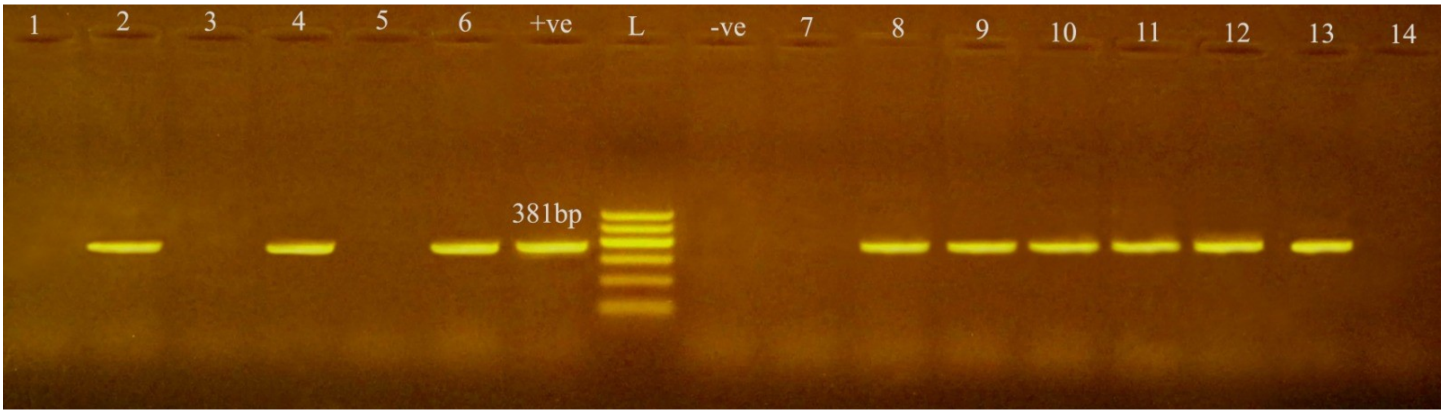

Fig. 1

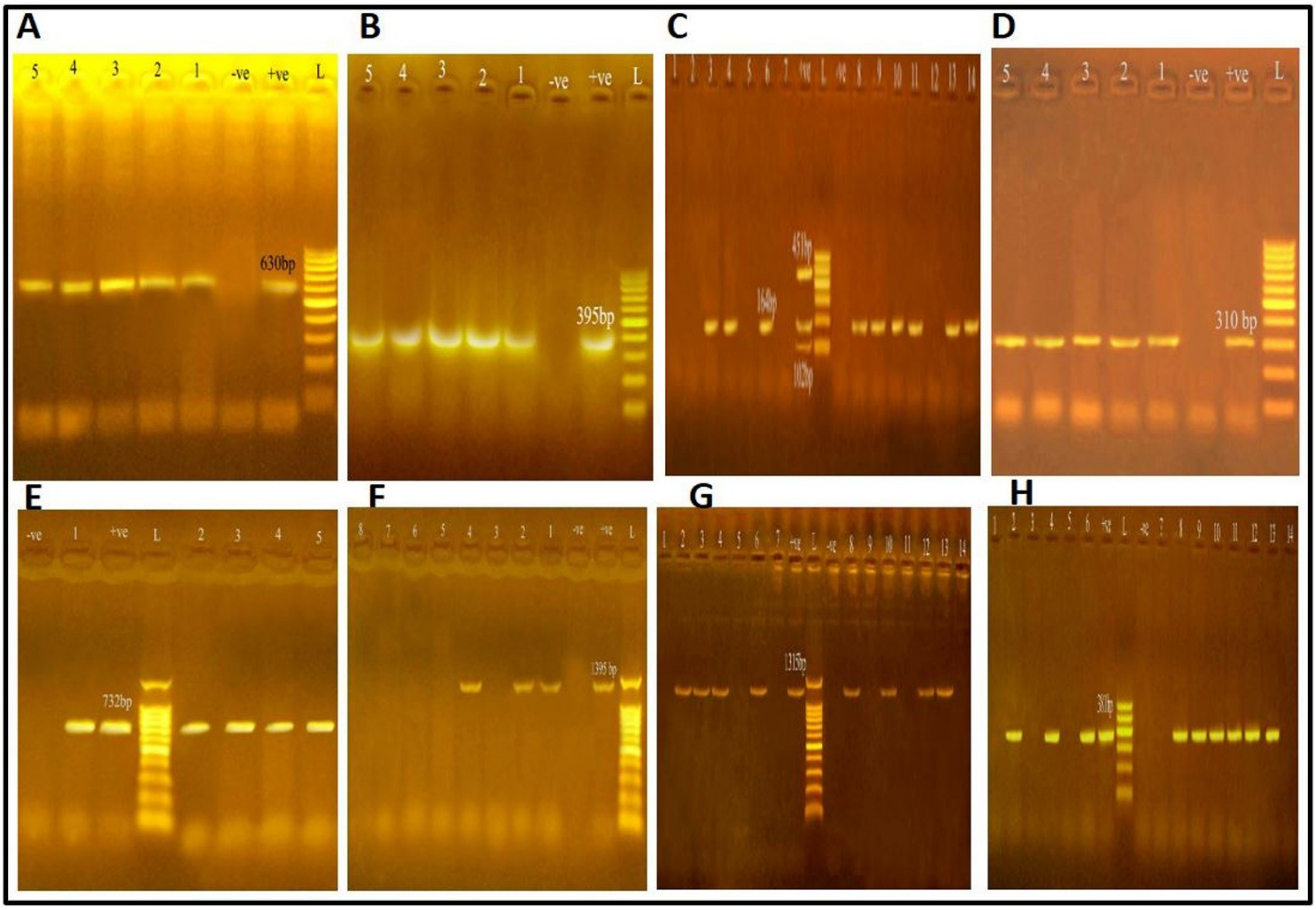
A
B
C
D
E
F
G
H
630bp
395bp
451bp
160bp
102bp
310 bp
732bp
1395 bp
1315bp
-ve
+ve
L
